# Impact of Baseline BMI on Glycemic Control and Weight Change with Metformin Monotherapy in Chinese Type 2 Diabetes Patients: Phase IV Open-Label Trial

**DOI:** 10.1371/journal.pone.0057222

**Published:** 2013-02-28

**Authors:** Linong Ji, Hongmei Li, Xiaohui Guo, Yan Li, Renming Hu, Zhengying Zhu

**Affiliations:** 1 Department of Endocrinology and Metabolism, Peking University People’s Hospital, Beijing, China; 2 Clinical Research, China Research & Development, Sino-American Shanghai Squibb Pharmaceutical Ltd, Shanghai, China; 3 Department of Endocrinology, Peking University First Hospital, Beijing, China; 4 Department of Endocrinology, Sun Yat-Sen Memorial Hospital, Sun Yat-Sen University, Guangzhou, China; 5 Department of Endocrinology and Metabolism, Huashan Hospital, Fudan University, Shanghai, China; 6 Clinical Research, China Research & Development, Bristol-Myers Squibb, Shanghai, China; Brigham & Women’s Hospital, and Harvard Medical School, United States of America

## Abstract

**Background:**

Differences exist between treatment recommendations regarding the choice of metformin as first-line therapy for type 2 diabetes patients according to body mass index (BMI). This study compared the efficacy of metformin monotherapy among normal-weight, overweight, and obese patients with newly diagnosed type 2 diabetes.

**Methods:**

In this prospective, multicenter, open-label study in China, patients aged 23–77 years were enrolled 1∶1:1 according to baseline BMI: normal-weight (BMI 18.5−23.9 kg/m^2^; n = 125); overweight (BMI 24.0−27.9 kg/m^2^; n = 122) or obese (BMI ≥28 kg/m^2^; n = 124). Extended-release metformin was administered for 16 weeks (500 mg/day, up-titrated weekly to a maximum 2,000 mg/day). The primary efficacy endpoint was the effect of baseline BMI on glycemic control with metformin monotherapy, measured as the change from baseline in glycosylated hemoglobin (HbA_1c_) at week 16 compared among BMI groups using ANCOVA. Other endpoints included comparisons of metformin’s effects on fasting plasma glucose (FPG), lipid levels and body weight.

**Results:**

Mean HbA_1c_ decreases at week 16, adjusted for baseline values, were –1.84%, –1.78% and –1.78% in normal-weight, overweight and obese patients, (*P* = 0.664); body weight decreased by 2.4%, 3.9% and 3.5%, respectively. FPG levels decreased similarly over time in all BMI groups (*P* = 0.461) and changes from baseline in high-density lipoprotein cholesterol (HDL-C) and low-density lipoprotein cholesterol (LDL-C) did not differ significantly among BMI groups at week 16 (*P* = 0.143 and 0.451, respectively).

**Conclusions:**

Baseline BMI had no impact on glycemic control, weight change or other efficacy measures with metformin monotherapy. These data suggest that normal-weight type 2 diabetes patients would derive the same benefits from first-line treatment with metformin as overweight and obese patients, and are not at increased risk of excess weight loss.

**Trial Registration:**

ClinicalTrials.gov NCT00778622

## Introduction

Metformin is an antihyperglycemic agent widely used in the treatment of type 2 diabetes. Because of its potent blood glucose-lowering efficacy, beneficial effects on body weight and lipid profiles, low risk of hypoglycemia with monotherapy, and its protective effect on the cardiovascular system [1], metformin is recommended as first-line antihyperglycemic treatment for type 2 diabetes in almost all international or national diabetes guidelines [2]–[7]. However, differences exist amongst guidelines regarding specific recommendations for the first-line use of anti-hyperglycemic agents, in terms of patients’ body mass index (BMI). For example, in diabetes treatment guidelines developed by the International Diabetes Federation (IDF) [8] and the Asian-Pacific Type 2 Diabetes Policy Group [4], metformin is recommended as the only first-line treatment in overweight and obese type 2 diabetes patients, while for those of normal body weight, metformin is one of several oral antihyperglycemic agents recommended as first-line therapy. However, there does not appear to be published evidence supporting these differences in recommendations with regard to the choice of metformin for type 2 diabetes patients on the basis of body weight.

In guidelines where body weight is a factor affecting the first-line choice of treatment, ‘overweight’ is defined as a BMI ≥25 kg/m^2^ [6]. However, a large proportion of Asian and Chinese patients with type 2 diabetes are of normal weight: a recently published pooled cross-sectional analysis of 39,794 diabetes patients from Asia (most of whom had type 2 diabetes), revealed that 64% had a BMI <25 kg/m^2^ [9]. One regional Chinese study showed that 59.2% of 521 diabetes patients in Hong Kong had a BMI <25 kg/m^2^ [10] and another showed that 36% of 4,160 patients in Shanghai had a BMI <24 kg/m^2^ [11].

Nevertheless, the efficacy and safety of metformin in normal-weight Chinese patients with type 2 diabetes have not been described. Although metformin is the first-line treatment recommended for normal-weight patients in the Chinese guidelines for the management of type 2 diabetes [3], physicians are concerned about a lack of effectiveness and the possibilities of excess weight loss in normal-weight patients this population.

Studies in Western populations have shown that the glycemic response to metformin is similar in obese and non-obese patients [12]–[15]. However, most of these were retrospective or observational studies, involved mainly Caucasians, and ‘non-obesity’ was defined as BMI <27 kg/m^2^ [14], <28 kg/m^2^ [15], or <30 kg/m^2^ [12]. Another retrospective study in Japanese type 2 diabetes patients showed that metformin had similar effects on glycosylated hemoglobin (HbA_1c_) levels in obese (defined as BMI ≥25 kg/m^2^) and non-obese (BMI <25 kg/m^2^) patients, with no significant differences in BMI change between study groups [16].

On the basis of this evidence, we hypothesized that metformin would have similar efficacy in type 2 diabetes patients, irrespective of baseline BMI. Hence, this prospective study was conducted in Chinese patients newly diagnosed with type 2 diabetes, to compare the effect of extended-release metformin monotherapy on glycemic control [measured by HbA_1c_ levels and fasting plasma glucose (FPG)], lipid levels, and body weight among normal-weight (defined here as BMI <24 kg/m^2^), overweight (BMI 24−27.9 kg/m^2^) and obese (BMI ≥28 kg/m^2^) patients.

## Materials and Methods

### Ethics Statement

Most study centers accepted the protocol that was approved by the central Ethics Committee (cEC) at the Peking University People’s Hospital, which was the central Institutional Review Board (IRB). Study centers that required it had the protocol approved by their own independent Ethics Committees (iEC); these included: Peking University First Hospital, Beijing Friendship Hospital, People’s Liberation Army Second Artillery Hospital, People’s Liberation Army 304 Hospital, Xinhua Hospital affiliated with Shanghai Jiao Tong University School of Medicine, and Sun Yat-Sen Memorial Hospital. The protocol was implemented in accordance with provisions of the Declaration of Helsinki and Good Clinical Practice guidelines. Freely given written informed consent had to be obtained from every patient or, in those situations where consent could not be given by patients, their legally acceptable representative, prior to clinical study participation, including informed consent for any screening procedures conducted to establish patient eligibility for the study.

### Study Design and Treatment

The protocol for this trial and supporting CONSORT checklist are available as supporting information; see Checklist S1 and Protocol S1.

This prospective, multicenter, Phase IV, three-arm, open-label study had two treatment periods: a screening period (up to 7 days) and a treatment period (metformin treatment for 16 weeks).

Patients were screened for eligibility, then enrolled into one of three study arms in a 1∶1:1 ratio according to their baseline BMI: normal-weight (BMI 18.5−23.9 kg/m^2^); overweight (BMI 24.0−27.9 kg/m^2^) or obese (BMI ≥28 kg/m^2^). The BMI of 18.5–23.9 kg/m^2^ used as the definition of normal weight in this study was based on recommendations by the Working Group on Obesity in China [17].

Metformin (metformin XR [Glucophage® XR], Bristol Myers Squibb) was administered to patients in all three groups from day 1 (baseline) as follows. The initial dose was 500 mg/day taken orally with the evening meal. After 7 days (week 1), the dose was up-titrated in increments of 500 mg weekly until the maximum daily dose of 2,000 mg/day was reached, unless intolerance or hypoglycemia was experienced. From week 4, the maximum daily dose was 2,000 mg/day if FPG was >7.0 mmol/L (126 mg/dL). If the FPG was >10.0 mmol/L (>180 mg/dL) at weeks 4, 8, or 12 and this level was confirmed at a repeated measurement after 1 week, the patient discontinued treatment.

Compliance based on study pill count was performed at each scheduled visit, which took place at day 1 (baseline) and at weeks 4, 8, 12 and 16 (±7 days for each visit). At each of these visits, patients were also given diet and lifestyle advice, and asked to record these changes in the patient diary provided; these were reviewed with the patient from week 4 onwards.

### Patient Eligibility

Patients were included in the study if they were aged 17−79 years old and Chinese Asian; had been diagnosed with type 2 diabetes within 6 months of enrollment; HbA_1c_ was 7.0−10.0%; and they were treatment-naïve for oral antidiabetic agents (i.e. had not received antidiabetic medication previously, or had received antidiabetic medication for ≤14 days and not within 1 month of enrollment).

Exclusion criteria were BMI ≥35 kg/m^2^ or <18.5 kg/m^2^; active liver disease and/or significant abnormal liver function, acute or chronic metabolic acidosis, including diabetic ketoacidosis; congestive heart failure defined as New York Heart Association class III/IV and/or left ventricular ejection fraction ≤40%; significant cardiovascular history within the past 6 months; severe retinopathy, persistent uncontrolled hypertension (systolic blood pressure ≥180 mm Hg, or diastolic blood pressure ≥105 mm Hg); severe chronic gastrointestinal disease; anemia; serum creatinine ≥1.5 mg/dL (133 µmol/L; males), ≥1.4 mg/dL (124 µmol/L; females); use of any other oral antidiabetic agents (including Chinese traditional medicine).

### Laboratory Assessments

Blood and urine samples were obtained during the scheduled visits for clinical laboratory evaluations. HbA_1c_ was tested at a central laboratory; other laboratory tests, such as hematology, serum chemistry (including fasting serum lipids), FPG, urinalysis and pregnancy tests, were carried out at local laboratories.

### Endpoints and Evaluations

The primary efficacy endpoint was the change from baseline in HbA_1c_ at week 16. HbA_1c_ levels were measured at the screening visit to obtain the baseline value, and at the week 16 visit or early termination visit. To avoid any potential impact of baseline HbA_1c_ levels on the findings, analysis of metformin’s effect on HbA_1c_ levels at week 16 was performed after adjustment for baseline HbA_1c_ values – this was a deviation from the original protocol, in which this adjustment was not specified.

The secondary efficacy endpoints were: (i) the change from baseline in FPG levels over time, where FPG levels were measured at the screening visit, and at visits on day 1 (baseline) and weeks 4, 8, 12, 16 and/or the early termination visit; and (ii) changes from baseline in fasting lipids at week 16. Total cholesterol (TC), low-density lipoprotein cholesterol (LDL-C), high-density lipoprotein cholesterol (HDL-C), and triglyceride (TG) levels were measured at the same visits as for HbA_1c_.

Other endpoints included the change from baseline over time in BMI and weight, in waistline, hipline and waist/hip ratio at week 16, and the percentage of subjects who reached HbA_1c_ levels of <7% at week 16. Weight was measured at each visit and percentage reduction in body weight was calculated as the change in body weight at the relevant time point divided by the baseline body weight, multiplied by 100. Waist and hip circumferences were measured at day 1 and the week 16 or early termination visits.

#### To determine safety, adverse events were evaluated at all visits from baseline onwards

Adverse events were classified by system organ class and preferred term according to the Medical Dictionary for Regulatory Activities (MedDRA version 14.0). An adverse event was defined as any new untoward medical occurrence or worsening of a pre-existing medical condition in a patient who was given an investigational (medicinal) product, and that did not necessarily have a causal relationship with this treatment. A treatment-emergent adverse event was defined as an event that occurred during the treatment period, i.e. starting on or after the date of first study treatment administration. Drug-related adverse events were those judged by the investigator to be either certainly, probably, possibly, not likely, or not related to study medication. A serious adverse event was defined as any untoward medical occurrence that occurred at any dose: including death, life-threatening conditions, hospitalization or causing prolongation of existing hospitalization, persistent or significant disability/incapacity; congenital anomaly/birth defect, or an important medical event that may jeopardize the subject or may require intervention.

Safety-related laboratory measurements were performed at screening and at week 16 or the early termination visit. Patients were instructed to inform the investigator as soon as they noticed any symptoms that might be associated with lactic acidosis, and treatment was to be withdrawn until the cause was established.

### Statistical Analyses

The sample size was calculated to allow the estimation of mean change from baseline in HbA_1c_ at week 16 with sufficient precision in each of the three baseline BMI subgroups. Assuming the standard deviation for the changes from baseline in HbA_1c_ was 1.0 across the planned baseline BMI subgroups, 97 patients in a single subgroup would have been sufficient to estimate the mean change in HbA_1c_ with a precision of 0.20% within the subgroup. Given the number of baseline BMI subgroups and that no correction for reasons of multiplicity were made to the 95% CI within each BMI subgroup, the total sample size had to be at least 291 for this study. The sample size calculation was performed using Query Advisor® v6.0 statistical software (Statistical Solutions, Saugus, Massachusetts).

Descriptive statistics were used to provide an overview of the primary and secondary efficacy outcomes and the safety results according to BMI group.

For the primary efficacy endpoint analysis, the last observation carried forward (LOCF) imputation and the observed case were used. The LOCF dataset was the primary dataset for the efficacy analyses. Point estimates and 95% CIs were calculated for least squares (LS) means of change from baseline at week 16 after adjustment for baseline HbA_1c_ levels. To compare the treatment effect on HbA_1c_ among the three BMI subgroups, the analysis of covariance (ANCOVA) model was used, with change from baseline in HbA_1c_ at week 16 as the dependent variable, BMI subgroup as the fixed main effect, and baseline HbA_1c_ as the covariate. To examine the relationship between baseline BMI and secondary endpoints, ANCOVA was used, with change from baseline in FPG or fasting lipids as dependent variables, BMI group as the main effect, and baseline FPG or lipids as the covariates. The analysis of variance (ANOVA) model was used with either baseline BMI, waistline, hipline, total dose, or duration of exposure as the dependent variables and BMI subgroup as the independent variable. All contrasts were interpreted at a two-sided 5% significance level, without adjustment for multiplicity.

Pairwise comparisons between BMI subgroups for continuous efficacy variables were made using the t test without multiplicity adjustment. All interpretations were based on a two-sided 5% significance level without correction for multiplicity. Adverse event data were summarized according to baseline BMI.

## Results

**Figure 1:**
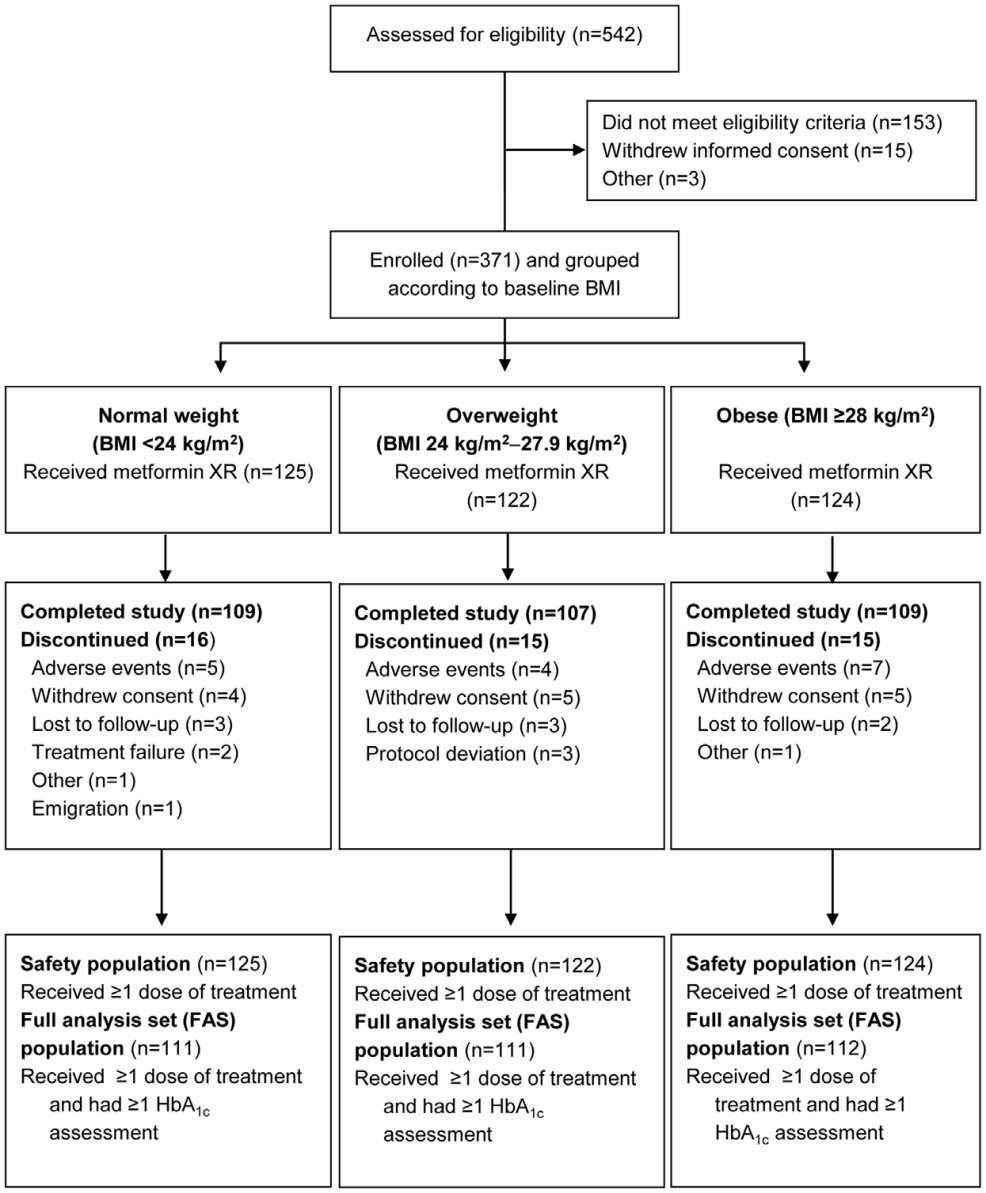
CONSORT flow diagram of patient disposition. BMI, body mass index.

This study (NCT00778622) was conducted at 20 hospitals in China between 19 November 2009 and 15 March 2011. A total of 371 patients aged 23−77 years were enrolled (Fig. 1). All these patients, who took at least one dose of study medication, were included in the safety analysis set or population; 334 patients who took one dose of study medication and had at least one post-baseline HbA_1c_ assessment were included in the full analysis set (FAS) population. ‘Treatment failure’ was cited as a reason for study discontinuation by two participants (Fig. 1), in accordance with the study protocol, which stated that patients had to discontinue study treatment if FPG levels were >10.0 mmol/L (>180 mg/dL) at visits at week 4 or afterwards (week 8, week 12), and if this was confirmed by a repeated measurement one week later.

Patient demographics and baseline disease characteristics are summarized in Table 1. Although a statistically significant difference was seen among groups in terms of gender in the baseline FAS population (P = 0.030), this variable was not included in the multivariable models because gender was not expected to have an impact on efficacy, according to previous findings [18], [19]. Of the total study population, 98.5% were Han Chinese. The patients’ weight ranged from 43.5 kg in the normal-weight group to 118.0 kg in the obese group. BMI ranged from 18.5–40.4 kg/m^2^ because one patient with a BMI of 40.4 kg/m^2^ was mistakenly enrolled in the study. He was allowed to participate in the study because the investigators felt that he would benefit from receiving metformin treatment. This patient met the FAS population criteria and he was included in the FAS dataset.

**Table 1:** Patient demographics and baseline disease characteristics according to baseline BMI.

Parameter	Normal (n = 111)	Overweight (n = 111)	Obese (n = 112)	*P* value
Male, n (%)	53 (47.7)	72 (64.9)	67 (57.5)	0.030†
Age (years), mean (SD)	51.8 (10.1)	52.0 (9.1)	52.3 (11.3)	0.930‡
Height (m), mean (SD)	1.65 (0.08)	1.66 (0.09)	1.66 (0.09)	0.287‡
Weight (kg), mean (SD)	61.3 (7.4)	72.3 (8.4)	83.4 (11.9)	<0.0001‡
BMI (kg/m^2^ ), mean (SD)	22.6 (1.3)	26.0 (1.2)	30.13 (2.2)	<0.0001‡
Waist/hip ratio, mean (SD)	0.92 (0.09)	0.94 (0.08)	0.95 (0.08)	0.016‡
Age at diagnosis of diabetes (years), mean (SD)	51.7 (10.1)	51.9 (9.1)	52.4 (11.3)	NC
Duration of diabetes at baseline (days), mean (SD)	37.9 (44.7)	36.1 (47.1)	37.0 (46.1)	NC
HbA_1c_ (%), mean (SD)	8.50 (0.84)	8.38 (0.85)	8.26 (0.77)	0.081‡
FPG (mmol/L), mean (SD)	8.28 (1.96)	8.48 (1.81)	8.29 (1.68)	0.935§
TC (mmol/L), mean (SD)	5.15 (1.06)	5.01 (1.04)	4.95 (1.01)	NC
LDL-C (mmol/L), mean (SD)	3.21 (0.74)	3.03 (0.94)	3.02 (0.83)	NC
HDL-C (mmol/L), mean (SD)	1.18 (0.25)	1.13 (0.39)	1.12 (0.25)	NC
TG (mmol/L), mean (SD)	1.87 (2.28)	2.63 (2.46)	2.34 (2.97)	NC
**Frequency of medical history events reported in >4% of BMI subgroups, n (%)**				
Endocrine/metabolism other than type 2 diabetes	36 (32.4)	41 (36.9)	36 (32.1)	NC
Disease of cardiovascular system	26 (23.4)	31 (27.9)	47 (42.0)	NC
History of smoking	10 (9.0)	25 (22.5)	24 (21.4)	NC
Surgery	8 (7.2)	7 (6.3)	15 (13.4)	NC
Disease of liver and gallbladder (including hepatitis B)	5 (4.5)	6 (5.4)	14 (12.5)	NC

BMI, body mass index; SD, standard deviation; NC, not calculated; HbA_1c,_ glycosylated hemoglobin; FPG, fasting plasma glucose; TC, total cholesterol; LDL-C, low-density lipoprotein cholesterol; HDL-C, high-density lipoprotein cholesterol; TG, triglycerides.

† P values are from Fisher’s exact tests.

‡ P values are from analysis of variance.

§ P values are from analysis of covariance.

As expected, there was a significant difference in baseline BMI among the three groups (*P*<0.0001 by ANOVA). The mean duration of type 2 diabetes at the time of commencing metformin treatment was very similar among BMI groups, at around 6 weeks.

Mean baseline HbA_1c_ levels were similar, and slightly lower in the overweight (8.38%) and obese patients (8.26%) than in the normal-weight patients (8.50%). Baseline FPG and fasting lipid levels were similar among the BMI groups, except TG levels, which were higher in the overweight and obese groups than in the normal-weight group.

### Efficacy

With regard to the primary endpoint, i.e. change from baseline in HbA_1c_ at week 16, glycemic control by metformin was not affected by baseline BMI. No statistically significant differences were observed between changes from baseline in HbA_1c_ among the three BMI subgroups when adjusted for baseline HbA_1c_ level (*P* = 0.664 by ANCOVA) (Table 2). LS mean changes from baseline were –1.84%, –1.73%, and –1.78% in the normal-weight, overweight and obese patient groups, respectively (*P* = 0.664 by ANCOVA).

**Table 2:** Week 16 values and change from baseline for efficacy parameters in the FAS population.

Parameter	Normal (n = 111)	Overweight (n = 111)	Obese (n = 112)	*P* value	
**HbA_1c_ (%)**					
Mean (SD)	6.56 (0.64)	6.60 (0.60)	6.58 (0.58)	0.664†	
LS mean change* (95% CI)	–1.84 (–1.95, –1.73)	–1.78 (–1.89, –1.67)	–1.78 (–1.89, –1.67)	0.664†	
**FPG (mmol/L)**					
Mean (SD)	6.54 (1.15)	6.68 (1.42)	6.42 (1.19)	0.461‡	
Mean change (SD)	–1.98 (1.79)	–2.17 (2.12)	–2.14 (2.03)	0.461‡	
95% CI	–2.32, –1.64	–2.57, –1.77	–2.52, –1.76		
**TC (mmol/L)**					
Mean (SD)	4.77 (0.77)	4.95 (1.01)	4.80 (0.93)	0.031‡	
Mean change (SD)	–0.39 (0.92)	–0.05 (0.96)	–0.14 (0.82)	0.03‡	
95% CI	–0.56, –0.21	–0.23, 0.13	–0.30, 0.02		
**LDL-C (mmol/L)**					
Mean (SD)	2.91 (0.61)	2.88 (0.85)	2.87 (0.77)	0.451‡	
Mean change (SD)	–0.31 (0.57)	–0.14 (0.66)	–0.18 (0.63)	0.451‡	
95% CI	–0.42, –0.20	–0.27, –0.02	–0.30, –0.06		
**HDL-C (mmol/L)**					
Mean (SD)	1.23 (0.26)	1.14 (0.30)	1.14 (0.28)	0.143‡	
Mean change (SD)	0.06 (0.20)	0.02 (0.34)	0.03 (0.20)	0.143‡	
95% CI	0.02, 0.09	–0.04, 0.09	–0.01, 0.07		
**TG (mmol/L)**					
Mean (SD)	1.71 (0.97)	2.90 (3.89)	2.27 (1.67)	0.021‡	
Mean change (SD)	–0.17 (2.07)	0.26 (3.32)	–0.04 (2.20)	0.021‡	
95% CI	–0.56, 0.22	–0.37, 0.90	–0.47, 0.39		
**BMI (kg/m^2^)**					
Mean (SD)	22.01 (1.53)	25.04 (1.51)	29.09 (2.31)	NC	
Mean change (SD)	–0.54 (0.84)	–1.00 (1.06)	–1.04 (1.11)	NC	
**Waist/hip ratio**					
Mean (SD)	0.90 (0.07)	0.94 (0.08)	0.94 (0.08)	<0.0001§	
Mean change (SD)	–0.02 (0.09)	–0.01 (0.10)	–0.01 (0.08)	0.446§	

HbA_1c_, glycosylated hemoglobin; FPG, fasting plasma glucose; BMI, body mass index; FAS, full analysis set; LS, least squares; SD, standard deviation; CI, confidence interval; TC, total cholesterol; LDL-C, low-density lipoprotein cholesterol; HDL-C, high-density lipoprotein cholesterol; TG, triglycerides; NC, not calculated. *LS means of change from baseline are adjusted by baseline HbA_1c_.

† *P* values are from analysis of covariance (ANCOVA) with BMI group as an effect and the baseline HbA_1c_ value as a covariate.

‡ *P* values are from ANCOVA.

§ *P* values are from analysis of variance.

**Figure 2:**
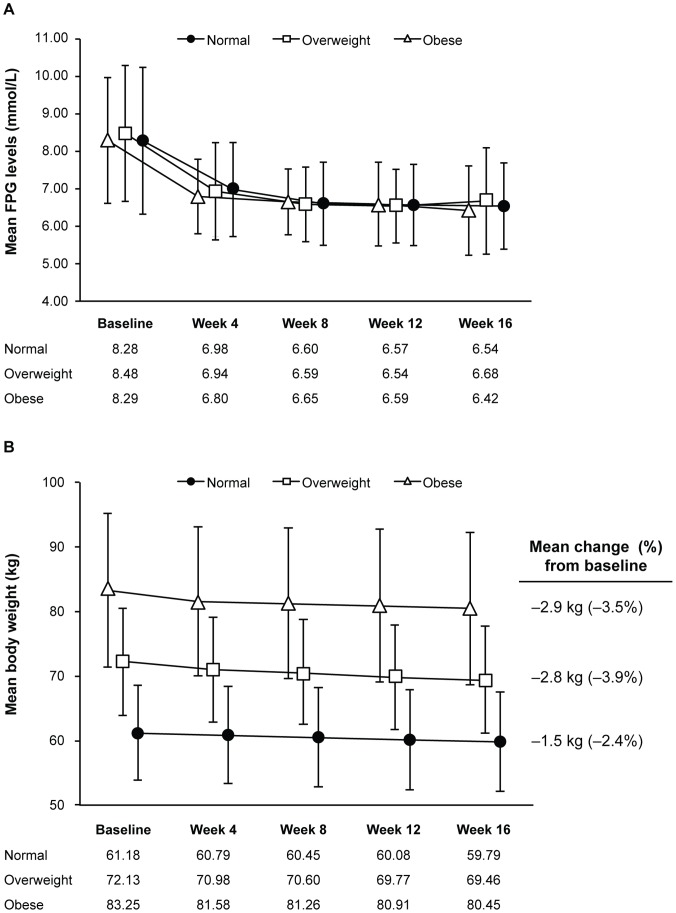
Changes over time according to baseline BMI in the full analysis set population. (A) Mean fasting plasma glucose (FPG) levels; (B) Mean body weight. Error bars represent standard deviation (SD).

Mean FPG levels decreased similarly over time in all BMI groups during 16 weeks’ treatment with metformin (Fig. 2A, Table 2). No statistically significant difference among the three BMI subgroups was found between changes from baseline in FPG levels at any time point (*P* = 0.461 by ANCOVA).

A total of 77.8% of the total study population reached an HbA_1c_ target of <7.0% [20]: 77.5% of the normal-weight group, 81.1% of the overweight group and 75.0% of the obese group.

Changes from baseline in lipid levels can be seen in Table 2. At week 16, the modest decreases in TC from baseline were significantly different among BMI groups (*P* = 0.031 by ANCOVA), with the greatest decrease in mean TC levels being observed in the normal-weight group (*P* = 0.008 versus the overweight group and *P* = 0.042 versus the obese group by pairwise comparison). A similar trend in decrease in mean LDL-C levels was seen at week 16, although there was no significant difference among BMI groups (*P* = 0.451 by ANCOVA). Once again, the decrease was greatest in the normal-weight group, although the difference was not significantly different from either of the other two BMI groups (*P* = 0.053 versus the overweight group and *P* = 0.130 versus the obese group by pairwise comparison). There was also no statistically significant difference among BMI groups with regard to change in mean HDL-C levels (*P* = 0.143 by ANCOVA), which increased slightly in all groups.

At week 16, the changes in mean TG levels from baseline were significantly different among BMI groups (Table 2): –0.17 mmol/L in the normal-weight group, 0.26 mmol/L in the overweight group and –0.04 mmol/L in the obese group (*P* = 0.021 by ANCOVA). However, considering the significant differences amongst the baseline TG levels between groups and the large variability in baseline values (Table 1), the changes in TG levels at week 16 among different BMI groups should be interpreted with caution.

Patients’ weight decreased gradually but slightly over the treatment time (Fig. 2B), with the smallest percentage reduction in body weight (2.4%) being observed in the normal-weight group at week 16. The initial percentage reductions in body weight at week 4 were 0.9%, 1.8% and 2.0% in the normal, overweight and obese study groups, respectively. At week 12, the respective percentage reductions were 1.9%, 3.6% and 3.3%. These were close to the percentage reductions seen at week 16 (Fig. 2B). BMI decreases over treatment time mirrored the weight-loss trends in each BMI group, with greater mean decreases from baseline being observed in the overweight (3.7%) and obese subgroups (3.4%) than in the normal-weight group at week 16 (2.4%; Table 2).

At week 16, waistline measurements decreased from baseline by a mean (SD) of 2.2 (4.92) cm in the normal-weight group, 2.2 (5.92) cm in the overweight group and 2.3 (5.75) cm in the obese group. No statistically significant difference among the three BMI groups was found in waistline changes (*P* = 0.972 by ANOVA). Significant differences in mean waist/hip ratios were also not found for changes from baseline at week 16 among the three BMI subgroups (*P* = 0.446 by ANOVA; Table 2).

### Safety

In the safety population, 333 patients (89.8%) had a compliance of 80%–100%: 111 patients in the normal group, 109 patients in the overweight group, and 113 patients in the obese group.

There was no difference among BMI groups in terms of total exposure (i.e. dose) of metformin (*P* = 0.719 by ANOVA). The mean (SD) daily dose was 1,405.0 (400.5) mg/day in the normal-weight group; 1,421.7 (337.1) mg/day in the overweight group; and 1,446.5 (386.6) mg/day in the obese group. A maximum dose of 1,500 mg was taken by 68.0% of patients in the normal-weight group, 68.9% of patients in the overweight group and 67.7% of patients in the obese group. The 2,000 mg maximum dose was taken by 27.2% of normal-weight patients, 27.9% of overweight patients and 29.0% of obese patients. The mean (SD) duration of treatment was 107.1 (26.24) days in the normal group, 109.1 (22.53) days in the overweight group, and 108.0 (25.95) days in the obese group, with no statistically significant difference observed between duration of exposure among BMI subgroups (*P* = 0.831 by ANOVA).

A drug-related treatment-emergent adverse event was reported by 28.8% of patients in the safety population (Table 3). Two (0.5%) patients experienced serious adverse events; neither event (right upper lung cancer and hypertension, both of moderate severity) was judged to be treatment-related. The most common drug-related treatment-emergent adverse events were gastrointestinal disorders (reported by 21.3% overall). Adverse events were generally mild or moderate in intensity, with only 5 patients (1.3%) reporting severe or very severe drug-related treatment-emergent adverse events that were gastrointestinal in nature.

**Table 3:** Overview of adverse events in the safety population.

Parameter, n (%)	Normal (n = 125)	Overweight (n = 122)	Obese (n = 124)	Total (N = 371)
Patients reporting ≥1 adverse event	43 (34.4)	47 (38.5)	46 (37.1)	136 (36.7)
Treatment-emergent* adverse events	43 (34.4)	47 (38.5)	46 (37.1)	136 (36.7)
Drug-related treatment-emergent adverse events†	38 (30.4)	42 (34.4)	27 (21.8)	107 (28.8)
Adverse events leading to permanent discontinuation of study drug	5 (4.0)	5 (4.1)	8 (6.5)	18 (4.9)
Treatment-emergent serious adverse events	1 (0.8)	0 (0)	1 (0.8)	2 (0.5)
**Drug-related treatment-emergent adverse events reported by >1% of safety population**				
Diarrhea	17 (13.6)	12 (9.8)	4 (3.2)	33 (8.9)
Abdominal discomfort	2 (1.6)	13 (10.7)	2 (1.6)	17 (4.6)
Abdominal distension	6 (4.8)	7 (5.7)	2 (1.6)	15 (4.0)
Abdominal pain (upper)	2 (1.6)	3 (2.5)	2 (1.6)	7 (1.9)
Constipation	3 (2.4)	1 (0.8)	2 (1.6)	6 (1.6)
Nausea	2 (1.6)	5 (4.1)	4 (3.2)	11 (3.0)
Decreased appetite	2 (1.6)	1 (0.8)	2 (1.6)	5 (1.3)
Hyperuricemia	0	2 (1.6)	3 (2.4)	5 (1.3)
Lipid metabolism disorder	1 (0.8)	1 (0.8)	3 (2.4)	5 (1.3)
Hepatic function abnormal	2 (1.6)	1 (0.8)	1 (0.8)	4 (1.1)
Palpitations	4 (3.2)	3 (2.5)	1 (0.8)	8 (2.2)

* A treatment-emergent adverse event was defined as an adverse event that was reported on or after the date of first study drug administration.

† Drug-related adverse events were those judged by the investigator to be either certainly, probably, possibly, not likely related or not related to study medication.

The most common adverse event was diarrhea, reported by 8.9% overall. It was reported by a greater proportion of normal-weight patients than those in the other two groups (Table 3). The proportion of patients who had abnormal liver and kidney function parameters (alanine aminotransferase, aspartate aminotransferase, bilirubin) at baseline decreased after 16 weeks of treatment (from 7.0% to 5.9%; from 3.2% to 5.9%, and from 5.4% to 1.6%, respectively). No other clinically significant changes were observed in liver function and kidney function measurements in the safety population. Lactic acidosis was not reported by any patients in this study. One patient in the normal-weight group who received the 2,000 mg dose experienced hypoglycemia, which was judged to be treatment-related, but not severe, and did not lead to treatment discontinuation.

## Discussion and Conclusions

The data from this prospective study confirm that glycemic response to metformin monotherapy is similar among normal-weight (BMI <24 kg/m^2^), overweight and obese Chinese patients who have newly diagnosed type 2 diabetes.

Because the study was not randomized, analysis of metformin’s effect on HbA_1c_ levels at week 16 was performed after adjustment for baseline HbA_1c_ values to avoid any potential impact of baseline HbA_1c_ levels on the findings. No statistically significant differences between mean HbA_1c_ reductions from baseline were observed among the BMI groups (*P* = 0.664) at the end of this study. Mean FPG levels decreased similarly from baseline over time with metformin treatment, with no significant differences observed among BMI subgroups at any time point (*P* = 0.461). After 16 weeks’ metformin monotherapy, 77.5% of the normal-weight, 81.1% of the overweight and 75.0% of the obese study groups achieved the HbA_1c_ target of <7.0% [20].

With regard to fasting lipid levels, modest reductions in mean TC and LDL-C were observed at week 16 in all three BMI groups, as were slight increases in HDL-C. These week 16 mean changes from baseline were not significantly different among BMI groups for HDL-C and LDL-C (*P* = 0.143 and 0.451, respectively), but mean TC levels decreased to a greater extent in the normal-weight group than in the other two groups (*P* = 0.031). TG levels decreased slightly in the normal-weight and obese groups and increased in the overweight group, but the SD was large in all three groups, both at baseline and at week 16, suggesting that these findings are not clinically significant.

Importantly, although the mean weight (and hence BMI) decreased gradually but slightly over the study period in all three BMI groups, the smallest percentage decrease from baseline body weight (2.4%) was observed in the normal-weight group. These data suggest that physicians’ reluctance to prescribe metformin to normal-weight type 2 diabetes patients because of concerns about excessive weight loss appear be unfounded. Within the normal-weight group, four patients had BMI <18.5 kg/m^2^ at week 16. The week 16 BMI values of these four patients were 18.1 kg/m^2^, 18.2 kg/m^2^, 17.5 kg/m^2^, and 18.3 kg/m^2^, respectively. The corresponding percentage changes from baseline BMI were 6.2%, 2.2%, 16.7%, and 5.2%, respectively. With the exception of one patient, the body weight reduction for the other three patients was within the range of 2.0%–6.5%, and their absolute BMI values at week 16 were very close to the lower limit of normal BMI range. Considering the mean decrease from baseline in BMI was 2.4% in the normal-weight group, the one patient who had a BMI reduction of 16.7% might be considered to be an outlier.

Treatment with metformin was well tolerated overall, with no significant differences between adverse event rates observed among normal-weight, overweight and obese patients. The most common treatment-emergent adverse event was diarrhea, and the adverse event profile was similar to that reported previously for extended-release metformin, where a lower rate of gastrointestinal adverse events was observed with the extended-release formulation than with immediate-release metformin [21]. One patient in the normal-weight group experienced hypoglycemia, which was drug-related but not severe, and none experienced lactic acidosis.

Our findings are in good agreement with those of previous prospective [14] and retrospective [12], [13], [16] studies of metformin that show similar efficacy between obese and non-obese type 2 diabetes patients, although our study has the lowest cut-off point for defining overweight patients (i.e. BMI ≥24 kg/m^2^). In two studies that defined normal-weight patients as BMI <25 kg/m^2^ and overweight patients as BMI ≥25 kg/m^2^, HbA_1c_ was reduced by 1.46% [13] and 1.2% [16] in the normal-weight patients, and by 1.34% and 1.1% in the overweight patients, respectively, after 3–12 months of metformin treatment. Even after 12 months of treatment, no reduction in BMI was observed in the normal-weight cohort in the Japanese study [16].

The open-label study design and the absence of comparator agent might be regarded as study limitations. However, the study was designed to determine the impact of baseline BMI on metformin efficacy and to compare its glucose-lowering effect among different BMI groups, based on the assumption that the efficacy of metformin in type 2 diabetes patients is already well established. Hence, a placebo group was not included. The conclusions from the current study would most likely have been the same had a double-blind, placebo-controlled design been used.

One limitation was the relatively short duration of this study. However, data from long-term studies has shown that the glucose-lowering effect of metformin becomes stable after 3–4 months of treatment [16]. The reductions in HbA_1c_ levels observed in normal-weight and overweight patients in this study were similar to those observed over the same period in a longer-term study of obese (BMI ≥25 kg/m^2^) and non-obese Japanese type 2 diabetes patients. In that 3-year retrospective study, HbA_1c_ levels decreased the most during the first 3 months of treatment with an oral hypoglycemic agent in both obese and non-obese patients, and after 6 months HbA_1c_ levels reached a plateau for the remainder of the study. In another long-term, retrospective, observational study of metformin efficacy in obese versus non-obese patients [12], the duration of successful metformin monotherapy in patients who had a BMI <25 kg/m^2^ was 7.0 years, leading those authors to conclude that metformin might even be more effective in normal-weight patients with type 2 diabetes.

Another study limitation was that data on lifestyle and diet changes were not collected, so it is hard to determine to what extent they could have contributed to these findings.

In conclusion, baseline BMI had no impact on the efficacy of metformin as monotherapy in Chinese patients with newly diagnosed type 2 diabetes, or on weight changes in these patients, during 16 weeks of treatment. Our findings are relevant to those worldwide who develop guidelines or recommendations for type 2 diabetes treatment, because they suggest that the large existing population of normal-weight patients who have a BMI <24 kg/m^2^ would derive the same benefit from first-line treatment with metformin as overweight or obese patients, and are at no increased risk of excess weight loss.

## Supporting Information

Checklist S1 **CONSORT checklist.**
(DOC)

Protocol S1 **Clinical Trial Protocol.**
(PDF)
