# Supplementary material for: Impact of Baseline BMI on Glycemic Control and Weight Change with Metformin Monotherapy in Chinese Type 2 Diabetes Patients: Phase IV Open-Label Trial
Source: PLoS One. 2013 Feb 28;8(2):e57222. doi: 10.1371/journal.pone.0057222 (PMC3585309; doi:10.1371/journal.pone.0057222)
Supplement: Protocol S1 — Clinical Trial Protocol. (PDF) [file pone.0057222.s002.pdf]

Protocol S1: Clinical Trial Protocol

Protocol Number: CV138097
IND Number: NA
EUDRACT Number NA
Date: 26-Mar-2008
Revised Date 26-Jun-2009

Clinical Protocol CV138097

The Relationship between Baseline Body Weight and Glycemic Control following Metformin Extended-Release Tablets (Glucophage XR) Monotherapy in Chinese Patients with Newly Diagnosed Type 2 Diabetes

Revised Protocol Number: 01
Incorporates amendment(s) 01

Study Director / Central Medical Monitor
Hongmei Li
6F Fuxing Plaza 109 Yan Dang Road
P.R.China, Shanghai 200020
Telephone (office): 86-21-23218293
Fax: 86-21-53830389

Protocol Manager
Hannah Chen
6F Fuxing Plaza 109 Yan Dang Road
P.R.China, Shanghai 200020
Telephone (office): 86-21-23218240
Fax: 86-21-53860383

24-hr Emergency Telephone Number
Liyong Ren: 86 13817061932

Bristol-Myers Squibb Research and Development

This protocol contains information that is confidential and proprietary to Bristol-Myers Squibb

Replace all previous version(s) of the protocol with this revised protocol and please provide a copy of this revised protocol to all study personnel under your supervision, and archive the previous versions.

DOCUMENT HISTORY

Document	Date of Issue	Summary of Change
Original Protocol	01-Feb-2008	Not applicable
Amendment 01	26-Jun-2009	Changes on criteria of up-titration and discontinuation of study; Changes on Investigational products management; Clarification on specimen analysis by central lab or local lab; Logistic changes.
Revised Protocol 01	26-Jun-2009	Incorporates Amendment 01.

Draft v3.11 930026888

TABLE OF CONTENTS

TITLE PAGE 1

DOCUMENT HISTORY 2

TABLE OF CONTENTS 3

SYNOPSIS 8

1 INTRODUCTION AND STUDY RATIONALE 10

1.1 Research Hypothesis 10

1.2 Study Rationale 10

1.3 Overall Risk/Benefit Assessment 12

2 STUDY OBJECTIVES 14

2.1 Primary Objective 14

2.2 Secondary Objectives. 14

2.3 Exploratory Objective 14

3 ETHICAL CONSIDERATIONS 15

3.1 Good Clinical Practice 15

3.2 Institutional Review Board/Independent Ethics Committee 15

3.3 Informed Consent. 16

4 INVESTIGATIONAL PLAN 17

4.1 Study Design and Duration. 17

4.2 Study Population 18

4.2.1 Inclusion Criteria 18

4.2.2 Exclusion Criteria 19
4.2.3 Discontinuation of Subjects from Treatment 20
5 TREATMENTS. 21
5.1 Study Treatment 21
5.1.1 Investigational Product 22
5.1.2 Noninvestigational Product 22
5.1.3 Identification 22
5.1.4 Packaging and Labeling 22
5.1.5 Handling and Dispensing 23
5.2 Method of Assigning Subjects to a Treatment 23
5.3 Selection and Timing of Dose for Each Subject 24
5.3.1 Dose Modifications 24
5.4 Blinding/Unblinding 24
5.5 Concomitant Treatments 24
5.5.1 Prohibited and/or Restricted Treatments 24
5.5.2 Other Restrictions and Precautions 24
5.6 Treatment Compliance 25
6 STUDY ASSESSMENTS AND PROCEDURES 25
6.1 Flow Chart/Time and Events Schedule 25
6.1.1 Procedures by visit 29
6.1.2 Visit windows 29
6.1.3 Screening Period- Enrollment Visit (up to 7 days prior to Day 1): 30
6.1.4 Treatment Period: 30
6.1.4.1 Day 1 Visit: 30

Draft v3.11 930026888

6.1.4.2 Week 4, 8 and 12 Visits (Week 4, 8 and 12± 7 days): *31*

6.1.4.3 Week 16 Visit (Week 16 ± 7days) or Early termination Visit: *31*

6.2 Study Materials 32

6.3 Safety Assessments 32

6.3.1 Lactic acidosis: 32

6.3.2 Laboratory Assessments: 33

6.3.3 Vital Signs: 35

6.3.4 Electrocardiograms: 35

6.3.5 Physical Examinations: 35

6.3.6 Physical Measurements: 35

6.3.7 Concomitant Medications:. 35

6.4 Efficacy Assessments 36

6.4.1 Primary Efficacy Assessment 36

6.4.2 Secondary Efficacy Assessments 36

6.5 Pharmacodynamics Assessments 36

6.6 Pharmacogenomics Assessments 36

6.7 Outcomes Research Assessments 36

6.8 Other Assessments 36

7 ADVERSE EVENTS 37

7.1 Definitions 37

7.1.1 Serious Adverse Events 37

7.1.2 Nonserious Adverse Events 38

7.2 Assignment of Adverse Event Intensity and Relationship to Study Drug 38

7.3 Collection and Reporting 39

7.3.1 Serious Adverse Events 40
7.3.2 Handling of Expedited Safety Reports 41
7.3.3 Nonserious Adverse Events 42
7.4 Laboratory Test Abnormalities 42
7.5 Overdose 43
7.6 Pregnancy 43
7.6.1 Requirements for Pregnancy Testing 44
7.6.2 Reporting of Pregnancy 45
7.7 Other Safety Considerations 45
8 STATISTICAL CONSIDERATIONS 46
8.1 Sample Size Determination 46
8.2 Populations for Analyses 46
8.3 Endpoint Definitions 46
8.4 Analyses 47
8.4.1 Demographics and Baseline Characteristics 47
8.4.2 Safety Analyses 47
8.4.3 Efficacy Analyses 48
8.4.4 Pharmacokinetic Analyses 49
8.4.5 Pharmacodynamic Analyses. 49
8.4.6 Pharmacogenomic Analyses 49
8.4.7 Outcomes Research Analyses 50
8.4.8 Other Analyses 50
8.5 Interim Analyses 50
9 ADMINISTRATIVE SECTION 50
9.1 Compliance. 50

Draft v3.11 930026888

9.1.1 Compliance with the Protocol and Protocol Revisions 50
9.1.2 Monitoring 51
9.1.3 Investigational Site Training 51
9.2 Records Retention 51
9.2.1 Case Report Forms. 52
9.2.2 Study Drug Records 53
9.2.3 Investigational Product Records 53
9.3 Return and Destruction of Study Drug 54
9.3.1 Return of Study Drug 54
9.3.2 Destruction of Study Drug. 54
9.4 Publications 54
10 GLOSSARY OF TERMS 56
11 LIST OF ABBREVIATIONS 58
12 REFERENCES 61
APPENDIX 1 ADDITIONAL ETHICAL CONSIDERATIONS. . 63

Draft v3.11 930026888

SYNOPSIS

Clinical Protocol CV138097

Title of Study: Protocol CV138097: The Relationship between Baseline Body Weight and Glycemic Control following Metformin Extended-release Tablets (Glucophage XR) Monotherapy in Chinese Patients with Newly Diagnosed Type 2 Diabetes

Estimated Number of Study Centers and Countries/Regions: The study will be conducted in approximately 15 sites in China.

Study Phase: IV

Research Hypothesis: The baseline body mass index (BMI) has no effect on the response to Glucophage XR monotherapy in glycemic control in Chinese patients with newly diagnosed Type 2 Diabetes (T2DM).

Primary Objective: To investigate the effect of the baseline body mass index (BMI) on the response to Glucophage XR monotherapy in glycemic control in Chinese patients with newly diagnosed T2DM by examining the relationship between baseline BMI (defined according to the Chinese guidelines: Obese is defined as BMI≥28kg/m^2, overweight is defined as BMI ≥24kg/m^2 and <28kg/m^2, normal weight is defined as BMI≥18.5kg/m^2 and <24kg/m^2) and HbA1c reduction after a 16-week oral administration of Glucophage XR (calculated as Week 16 HbA1c - baseline HbA1c) in Chinese patients with newly diagnosed T2DM.

Study Design: Open label, 3-arm, multi-center trial in newly diagnosed Chinese T2DM subjects.

Duration of Study: The expected duration of the study, from the first subject, first visit through the last follow-up visit for the last subject, is approximately 12 months. The treatment period is 16 weeks.

Number of Subjects per Group: Approximately 111 subjects for each BMI group, a total of approximately 333 subjects will be enrolled.

Study Population: Chinese patients with Newly diagnosed T2DM (defined as T2DM diagnosed within 6 months prior to enrollment), aged 17 years or older and younger than 80 years, and oral antidiabetic agents naïve (defined as without receiving any anti-diabetic medication therapy before, or having received anti-diabetic medication ≤ 14 days but not received any antidiabetic medication within the last 1 month prior to enrollment) with HbA1c ≥ 7.0% and ≤10.0%.

Investigational Product(s), Dose and Mode of Administration, Duration of Treatment with Investigational Product(s):

Glucophage XR will be administered to subjects enrolled to the study for 16 weeks. The initial dose will be 500mg/day orally taken once daily with the evening meal, and then it will be up-titrated by increments of 500mg weekly to 1500mg/day unless intolerance or hypoglycemia occurs. At week 4 and afterwards, the maximum daily dose may be 2000mg/day (orally, once daily, with the evening meal), if FPG>7.0 mmol/L (126mg/dL).

Study Assessments and Primary Endpoints:

The primary efficacy endpoint of this study is the HbA1c change from baseline after a 16-week oral administration of Glucophage XR.

Draft v3.11 930026888

Statistical Methods:

The primary efficacy variable will be the change from baseline in HbA1c at Week16. Secondary efficacy variables will be the change from baseline in fasting plasma glucose, the relative changes from baseline in fasting lipids, and the changes from baseline in CRP, PAI-I, and Adiponectin.

All patients treated will be included in the analyses. The Last Observation Carried Forward (LOCF) data set includes data recorded at a given visit or, if no observation is recorded at that visit, data carried forward from the previous visit. Baseline data will not be carried forward or be averaged with on-treatment data to impute missing values for the LOCF data set. The Observed Case (OC) data set consists of the actual observations at each visit. Efficacy analyses will be performed using both the LOCF and OC data sets. The LOCF data set is the primary data set for the Efficacy Analyses.

Summary statistics will be provided for the primary and secondary efficacy variables for all patients treated as well as for each of the baseline BMI subgroups. The BMI subgroups are defined according to the Chinese guidelines: Obese is defined as BMI$\geq$28kg/m^2, overweight is defined as BMI $\geq$24kg/m^2 and <28kg/m^2, normal weight is defined as BMI$\geq$18.5kg/m^2 and <24kg/m^2.

Analysis of Variance (ANOVA) will be used with change from baseline in HbA1c at Week 16 as dependent variable and BMI subgroup (with categories “normal weight”, “obese”, and “overweight” defined on the basis of baseline BMI according to the Chinese guidelines) as main effect will be applied. The latter ANOVA analysis will be employed for contrasting the treatment effect in the normal weight subgroup versus the other two baseline BMI subgroups. Both contrasts will be interpreted at a two-sided 5% significance level, without correction for multiplicity.

To study the relationship between baseline BMI and change from baseline in HbA1c, and to assess potential predictive factors influencing response to Glucophage XR monotherapy, a multiple regression model with change from baseline in HbA1c at Week 16 as dependent variable will be performed. The covariates that will be used in the analysis are age at diagnosis of diabetes, duration of diabetes at time of commencing Glucophage XR treatment, gender, baseline HbA1c, baseline waist/hip ratio, waist circumference and baseline BMI.

Summary statistics will be provided per study week assessments were planned and will consist of number of patients assessed, sample mean value observed together with its associated standard error, sample mean change observed together with its associated standard error and 95% confidence interval. To summarize relative changes from baseline in fasting lipids [total cholesterol, low-density lipoprotein cholesterol (LDL-C), high-density lipoprotein cholesterol (HDL-C), and triglycerides (TG)], a log-transformation will be applied on the raw data prior to calculating summary statistics. Estimates of mean changes and associated standard errors and 95% confidence intervals calculated on the log scale will be back-transformed into the original scale.

The incidence of adverse events will be tabulated for all patients treated as well as by baseline BMI subgroup. AEs will be classified by Primary System Organ Class (SOC) and Preferred Term (PT) according to the Medical Dictionary for Regulatory Activities (MedDRA).

Draft v3.11 930026888

1 INTRODUCTION AND STUDY RATIONALE

1.1 Research Hypothesis

The baseline body mass index (BMI) has no effect on the response to Metformin extended-release (Glucophage XR) tablets monotherapy in achieving glycemic control in Chinese patients with newly diagnosed type 2 diabetes mellitus (T2DM).

1.2 Study Rationale

Metformin is a widely prescribed anti-diabetic drug, which has been demonstrated in the U.K. Prospective Diabetes Study (UKPDS) as efficacious as sulfonylureas in the treatment of obese diabetic patients (defined as >120% ideal body weight) in terms of glycemic control and is associated with less weight gain and fewer hypoglycemic episodes [1, 2]. Thus, metformin is well acknowledged as the first line therapy in obese individual with T2DM.

In 2006, the American Diabetes Association (ADA) and European Association for the Study of Diabetes (EASD) published a consensus to recommend metformin as the initial therapy of diagnosed T2DM and the therapy of each stage throughout the progress of T2DM as well, no matter the patients are obese/overweight or normal weight [3]. Furthermore, the similar recommendations are suggested in majority recent guidelines for Diabetes management, such as ADA (2007), the International Diabetes Federation (IDF) (2005) and NICE (2002), *etc.* [4-6]. The global guideline for T2DM from the IDF (2005) even concludes that the evidence on better prevention of arterial outcomes when using metformin in the overweight sub-study of UKPDS [1] supports the primary use of that drug in all overweight people with T2DM, and indeed probably in all people with T2DM [5].

In Asian-Pacific countries, the IDF Western Pacific region guidelines has endorsed and is consistent with the global IDF guidelines that metformin is recommended as first-line therapy in the obese and overweight, and is recommended as first-line therapy in nonobese patients in some countries [7].

Recently, an Australian retrospective study indicated that metformin was at least as efficacious in overweight (defined as body weight index (BMI) 26-30kg/m^2) and normal weight (BMI≤26kg/m^2) individuals with T2DM as it is in those who are obese (BMI≥30kg/m^2) [8]. Contemporaneously, a UK analysis in a population of 1701 T2DM patients receving metformin monotherapy showed the effect of BMI on the response to metformin was unlikely to be clinical relevant [9]. Furthermore, it has also been detected in a prospective study in nonobese (mean BMI 24-25 kg/m^2) T2DM patients, metformin treatment at the total maximum daily dose of 2 g vs repaglinide at the total maximum daily dose of 6 mg for 4 months achieved comparable decrease in HbA1c levels [10]. However, the efficacy and safety of metformin in Chinese normal weight T2DM patients is yet to be observed.

Currently, the definitions of normal weight (BMI<24kg/m^2), overweight (BMI≥24kg/m^2 and <28kg/m^2) and obese (BMI≥28kg/m^2) in China Guideline are different from the Global definitions (normal weight is BMI≤26kg/m^2, overweight is 26-30kg/m^2, and obese is BMI≥30kg/m^2). According to the Chinese definition of normal weight/overweight/obese, approximately 30% are normal weight based on the BMI distribution pattern in Chinese T2DM patients. However, in China Guidelines for Diabetes Management, metformin is recommended as the only first choice for obese and overweight T2DM patients. In normal weight T2DM patients, metformin is considered as one of the three choices of the first line therapy, the others are thiazolidinediones, sulfonylureas or glinides and alpha-glucosidase inhibitors [15]. Thus the China guidelines are different from the global ones and result in the actually first line use of metformin in normal weigh patients is only about 16% patients with T2DM in China. Therefore, it is very important to investigate whether the efficacy of metformin in normal weight T2DM patients is similar to its efficacy in overweight/obese T2DM patients.

Glucophage XR is a novel extended-release formulation of metformin, which is based on a dual hydrophilic polymer system named as GelShield diffusion system invented by BMS [11]. In the previous studies conducted in USA, it has shown that Glucophage XR has better tolerability and compliance and similar efficacy in T2DM treatment compared to the Glucophage immediate-release (IR) formulation [12-14]. In a double blind, placebo-controlled study by Fujioka, Glucophage XR significantly reduced HbA1c. Moreover, a clear dose-response relationship was evident at Glucophage XR doses up to

Draft v3.11 930026888

1500 mg , with treatment differences vs placebo of -0.6% (500 mg once daily), -0.7% (1000 mg once daily), and -1.0% (2000 mg once daily) in HbA1c [12]. However, it is still lack of efficacy and safety data of Glucophage XR in Chinese T2DM patients.

Therefore, we will investigate the efficacy and safety of Glucophage XR in Chinese normal weight (BMI≥18.5kg/m^2 and BMI<24kg/m^2), overweight (BMI≥24kg/m^2 and <28kg/m^2) and obese (BMI≥28kg/m^2) T2DM patients (The BMI stratification is according to the Chinese guideline 2007) who are newly diagnosed and have not been treated with any anti-diabetic medicines before. In the present study, our hypothesis is the baseline BMI has no effect on the response to Glucophage XR monotherapy in terms of glycemic control as measured by HbA1c levels in newly diagnosed Chinese patients with T2DM.

In the present study, the initial dose of Glucophage XR is proposed as 500mg QD with the evening meal, increasing upto 2000 mg QD as required. Selection of this doses range and titration method is based primarily on the label of Glucophage XR and the Phase III dose-ranging study of Glucophage XR in T2DM (CV138-036). In the CV138-036 study, Glucophage XR were administered to T2DM subjects at the doses range from 500 mg/day to 2000 mg/day (started from 500 mg/day with evening meal, and increased 500 mg per week, the maximum daily dose allowed was 2000 mg per day) . The results showed that Glucophage XR was superior to placebo in glucose control. The safety and the tolerability of Glucophage XR at the doses range from 500-2000mg/day have also been demonstrated in the previous studies [13, 14]

1.3 Overall Risk/Benefit Assessment

Glucophage XR is a widely prescribed anti-diabetic agent in USA, which has undergone extensive clinical studies. These clinical trials of Glucophage XR have shown its reliable efficacy. A 24-week, double-blind, placebo-controlled study of Glucophage XR taken once daily with the evening meal, was conducted in patients with T2DM who had failed to achieve glycemic control with diet and exercise (HbA1c 7.0%-10.0%, FPG 126-270 mg/dL). Patients entering the study had a mean baseline HbA1c of 8.0% and a mean baseline FPG of 176 mg/dL. After 12 weeks treatment, mean HbA1c had increased from baseline by 0.1% and mean FPG decreased from baseline by 2 mg/dL in the placebo group, compared with a decrease in mean HbA1c of 0.6% and a decrease in mean FPG of

23 mg/dL in patients treated with Glucophage XR 1000 mg once daily. Subsequently, the treatment dose was increased to 1500 mg once daily if HbA1c was ≥7.0% but <8.0% (patients with HbA1c ≥8.0% were discontinued from the study). At the final visit (24-week), mean HbA1c had increased 0.2% from baseline in placebo patients and decreased 0.6% with Glucophage XR.

A 16-week, double-blind, placebo-controlled, dose-response study of Glucophage XR, taken once daily with the evening meal or twice daily with meals, was conducted in patients with type 2 diabetes who had failed to achieve glycemic control with diet and exercise (HbA1c 7.0%-11.0%, FPG 7-15.6 mmol/L (126-280mg/dL)). Compared with placebo, improvement in glycemic control was seen at all dose levels of Glucophage XR (metformin hydrochloride extended-release tablets) and treatment was not associated with any significant change in weight.

Glucophage XR alone does not usually cause hypoglycemia, although it may occur when Glucophage XR is used in conjunction with oral sulfonylureas and insulin. When initiating combination therapy, the risks of hypoglycemia, its symptoms and treatment, and conditions that predispose to its development should be explained to patients and responsible family members.

There are also comprehensive clinical trials results on the safety profile of Glucophage XR. In worldwide clinical trials in patients with T2DM have been treated with Glucophage XR in placebo- and active-controlled studies. In placebo-controlled trials, 781 patients were administered Glucophage XR and 195 patients received placebo. Adverse reactions reported were greater than 5% in the Glucophage XR patients, and more common in Glucophage XR- than placebo-treated patients.

Diarrhea led to discontinuation of study medication in 0.6% of patients treated with Glucophage XR. Additionally, the following adverse reactions were reported in ≥1.0% - ≤5.0% of Glucophage XR patients and were more commonly reported with Glucophage XR than placebo: abdominal pain, constipation, distention abdomen, dyspepsia/heartburn, flatulence, dizziness, headache, upper respiratory infection, taste disturbance.

2 STUDY OBJECTIVES

2.1 Primary Objective

The primary efficacy objective of this study is to investigate the effect of the baseline body mass index (BMI) on the response to Glucophage XR monotherapy on glycemic control in Chinese patients with newly diagnosed T2DM by examining the relationship between baseline BMI (defined according to the Chinese guidelines: Obese is defined as BMI≥28kg/m^2, overweight is defined as BMI ≥24kg/m^2 and <28kg/m^2, normal weight is defined as BMI≥18.5kg/m^2 and <24kg/m^2) and mean HbA1c reduction after a 16-week oral administration of Glucophage XR (calculated as Week 16 HbA1c - baseline HbA1c) in Chinese patients with newly diagnosed T2DM.

2.2 Secondary Objectives

Secondary objectives are to study the effects of Glucophage XR monotherapy in Chinese patients with newly diagnosed T2DM at week16 for the following:

- the relationship between baseline BMI and fasting plasma glucose (FPG) reduction (calculated as Week16 FPG - baseline FPG).

- the relationship between baseline HbA1C and HbA1C reduction.

- the relationship between baseline waist circumference/waist hip ratio and HbA1C reduction.

- the relative changes from baseline in fasting lipids (Total cholesterol (Total-C), Low density lipoprotein-cholesterol (LDL-C), High density lipoproteincholesterol (HDL-C) and triglycerides (TG)) (calculated as Week16 fasting lipids - baseline fasting lipids).

2.3 Exploratory Objective

- the changes from baseline in CRP, PAI-1 and Adiponectin (calculated as Week16 CRP/PAI-1/Adiponectin - baseline CRP/PAI-1/Adiponectin) in first 111 patients who are enrolled by sites in Beijing, China.

Draft v3.11 930026888

3 ETHICAL CONSIDERATIONS

3.1 Good Clinical Practice

This study will be conducted in accordance with Good Clinical Practice (GCP), as defined by the International Conference on Harmonisation (ICH) and in accordance with the ethical principles underlying European Union Directive 2001/20/EC and the United States Code of Federal Regulations, Title 21, Part 50 (21CFR50).

The study will be conducted in compliance with the protocol. The protocol and any amendments and the subject informed consent will receive Institutional Review Board/Independent Ethics Committee (IRB/IEC) approval/favorable opinion prior to initiation of the study.

All potential serious breaches must be reported to BMS immediately. A serious breach is a breach of the conditions and principles of GCP in connection with the study or the protocol, which is likely to affect, to a significant degree, the safety or physical or mental integrity of the subjects of the study or the scientific value of the study.

Study personnel involved in conducting this study will be qualified by education, training, and experience to perform their respective task(s).

This study will not use the services of study personnel where sanctions have been invoked or where there has been scientific misconduct or fraud (eg, loss of medical licensure, debarment).

Systems with procedures that assure the quality of every aspect of the study will be implemented.

3.2 Institutional Review Board/Independent Ethics Committee

Before study initiation, the investigator must have written and dated approval/favorable opinion from the IRB/IEC for the protocol, consent form, subject recruitment materials/process (eg, advertisements), and any other written information to be provided to subjects. The investigator or sponsor should also provide the IRB/IEC with a copy of

Draft v3.11 930026888

the Investigator Brochure or product labeling, information to be provided to subjects and any updates.

The investigator or sponsor should provide the IRB/IEC with reports, updates and other information (eg, expedited safety reports, amendments, and administrative letters) according to regulatory requirements or institution procedures

3.3 Informed Consent

Investigators must ensure that subjects, or, in those situations where consent cannot be given by subjects, their legally acceptable representative, are clearly and fully informed about the purpose, potential risks, and other critical issues regarding clinical studies in which they volunteer to participate. Freely given written informed consent must be obtained from every subject or, in those situations where consent cannot be given by subjects, their legally acceptable representative, prior to clinical study participation, including informed consent for any screening procedures conducted to establish subject eligibility for the study.

The rights, safety, and well-being of the study subjects are the most important considerations and should prevail over interests of science and society.

Subjects unable to give their written consent (eg, stroke patients, or subjects with severe dementia) may only be enrolled in the study with the consent of a legally acceptable representative. The subject must also be informed about the nature of the study to the extent compatible with the subjects' understanding, and should they become capable, personally sign and date the consent form as soon as possible. The explicit wish of a subject unable to give his or her written consent, who is capable of forming an opinion and assessing this information to refuse participation in, or to be withdrawn from, the clinical study at any time should be considered by the investigator.

Appendix 1 contains BMS procedures on obtaining informed consent from subjects, or, in those situations where consent cannot be given by subjects, their legally acceptable representative prior to participating in a clinical study. Procedures are described for all subjects, including those who are unable to give informed consent. The relevant

procedures must be used whenever they are applicable (see subject selection criteria in Sections 4.2.1 and 4.2.2).

Before any study procedures are performed, subjects will have the details of the study described to them, and they will be given a written informed consent document to read. Then if subjects consent to participate in the study, they will indicate that consent by signing and dating the informed consent document in the presence of study personnel.

4 INVESTIGATIONAL PLAN

4.1 Study Design and Duration

The study is designed as an open-label, with 16 weeks Glucophage XR treatment duration trial, to investigate the effect of the baseline body mass index (BMI) on the response to Glucophage XR monotherapy in glycemic control in Chinese patients with newly diagnosed T2DM by examining the relationship between baseline BMI and HbA1c reduction after a 16-week oral administration of Glucophage XR (calculated as Week 16 HbA1c - baseline HbA1c) in Chinese patients with newly diagnosed T2DM.

The expected duration of the study, from first subject, first visit through the last visit for the last subject is approximately 12 months.

Based on their baseline BMI, eligible subjects will be enrolled to the 3 BMI subgroups in a 1:1:1 ratio. The BMI subgroups are defined according to the Chinese guideline 2007: Obese is defined as BMI≥28kg/m^2, overweight is defined as BMI ≥24kg/m^2 and <28kg/m^2, normal weight is defined as BMI≥18.5kg/m^2 and <24kg/m^2. Each subgroup will contain 111 subjects, including 10% of drop-out rate.

Glucophage XR will be administered to subjects enrolled to the study for 16 weeks. The initial dose will be 500mg/day orally taken once daily with the evening meal, and then it will be up-titrated by increments of 500mg weekly to 1500mg/day unless intolerance or hypoglycemia occurs. At week 4 and afterwards, the maximum daily dose may be 2000mg/day (orally, once daily, with the evening meal), if FPG>7.0mmol/L (126mg/dL) [15].

4.2 Study Population

For entry into the study, the following criteria MUST be met.

4.2.1 Inclusion Criteria

1) All subjects must be willing to provide **signed Written Informed Consent**
2) Age≥ 17 and <80 years, race: Chinese Asian
3) Newly diagnosed T2DM (defined as T2DM diagnosed within 6 months prior to enrollment)
4) Oral antidiabetic agents naïve (defined as without receiving any anti-diabetic medication therapy before, or having received anti-diabetic medication ≤ 14 days but not received any antidiabetic medication within the last 1 month prior to enrollment)
5) HbA1c ≥ 7.0% and ≤10.0%
6) Women of childbearing potential (WOCBP) must be using an adequate method of contraception to avoid pregnancy throughout the treatment period of the study or for 2 weeks after the last dose of study medication, whichever is longer, in such a mamer that the risk of pregnancy is minimized.

WOCBP include any female who has experienced menarche and who has not undergone successful surgical sterilization (hysterectomy, bilateral tubal ligation, or bilateral oophorectomy) or is not postmenopausal (defined as amenorrhea ≥ 12 consecutive months; or women on hormone replacement therapy [HRT] with documented serum follicle stimulating hormone [FSH] level > 35 mIU/mL). Even women who are using oral contraceptives, other hormonal contraceptives (vaginal products, skin patches, or implanted or injectable products), or mechanical products such as an intrauterine device or barrier methods (diaphragm, condoms, spermicides) to prevent pregnancy, or are practicing abstinence or where their partner is sterile (eg, vasectomy) should be considered to be of childbearing potential.

WOCBP must have a negative serum or urine pregnancy test (minimum sensitivity 25 IU/L or equivalent units of HCG) within 72 hours prior to the start of investigational product.

Draft v3.11 930026888

4.2.2 Exclusion Criteria

1) **Sex and Reproductive Status**
   a) WOCBP who are **unwilling or unable** to use an acceptable method to avoid pregnancy for the entire study period.
   b) Women who are pregnant or breastfeeding
   c) Women with a positive pregnancy test on enrollment or prior to investigational product administration.

2) **Target Disease Exceptions**
   a) BMI≥35 Kg/m^2 or BMI <18.5 Kg/m^2
   b) HbA1c>10.0% or <7.0%

3) **Medical History and Concurrent Diseases**
   a) Renal disease or renal dysfunction which may also result from conditions such as cardiovascular collapse (shock), acute myocardial infarction, and septicemia
   b) Active liver disease and/or significant abnormal liver function defined as ALT and/or AST > 1.5 X ULN and/or total bilirubin > 2 X ULN
   c) Acute or chronic metabolic acidosis, including diabetic ketoacidosis, with or without coma
   d) Congestive heart failure defined as New York Heart Association (NYHA) class III or IV and /or left ventricular ejection fraction ≤40%
   e) Significant cardiovascular history with the past 6 months defined as: myocardial infarction, coronary angioplasty or bypass graft(s), valvular disease or repair, unstable angina pectoris, transient ischemic attack, cerebrovascular accident or any findings in Electrocardiograms reports which in the opinion of the investigator are clinical significant.
   f) Severe retinopathy, persistent uncontrolled hypertension (SBP≥180mmHg, or DBP≥105mmHg)
   g) Severe chronic gastrointestinal disease
   h) History of alcohol abuse or illegal drug abuse within the past 12 months
   i) Diagnosed anemia

4) **Physical and Laboratory Test Findings**
   a) Creatine kinase ≥3 X ULN
   b) Serum creatinine ≥1.5 mg/dL(133μmol/L) [males], ≥1.4 mg/dL(124 μmol/L)[females]

Draft v3.11 930026888

c) ALT and/or AST > 1.5 X ULN and/or total bilirubin > 2 X ULN
d) Hemoglobin <12g/dL [males], <11g/dL [females]

5) Allergies and Adverse Drug Reactions

a) Known hypersensitivity to metformin hydrochloride

6) Prohibited Treatments and/or Therapies

a) Use of any other oral antidiabetic agents (including Chinese traditional medicine) or insulin together with Glucophage XR
b) Systemic use of glucocorticoids (excluding topical and inhaled steroids), and use of β-blockers, diuretic, phenothiazines, thyroid products, estrogens, oral contraceptives, phenytoin, nicotinic acid, sympathomimetics, nifedipine and isoniazid is necessary and can not be replaced by any other treatment
c) Participation in a clinical study involving an investigational drug or device during the last 90 days
d) Donation of blood, plasma or platelets within the past 3 months

7) Other Exclusion Criteria

a) Prisoners or subjects who are involuntarily incarcerated
b) Subjects who are compulsorily detained for treatment of either a psychiatric or physical (eg, infectious disease) illness
c) Subjects decline to participate

Eligibility criteria for this study have been carefully considered to ensure the safety of the study subjects and to ensure that the results of the study can be used. It is imperative that subjects fully meet all eligibility criteria.

4.2.3 Discontinuation of Subjects from Treatment

Subjects MUST discontinue investigational product (and noninvestigational product at the discretion of the investigator) for any of the following reasons:

Subjects MUST discontinue study treatment (investigational or noninvestigational treatment) for any of the following reasons:

Draft v3.11 930026888

- Withdrawal of informed consent (subject's decision to withdraw for any reason)
- Any clinical adverse event (AE), laboratory abnormality or intercurrent illness which, in the opinion of the investigator, indicates that continued participation in the study is not in the best interest of the subject
- Pregnancy (see Section 7.6.2)
- Termination of the study by Bristol-Myers Squibb (BMS)
- Loss of ability to freely provide consent through imprisonment or involuntarily incarceration for treatment of either a psychiatric or physical (eg, infectious disease) illness
- FPG level > 10.0 mmol/L (>180 mg/dL) at visits Week 4 or afterwards (Week 8, Week 12) and confirmed at a repeated measurement in one week
- Subjects whose BMI decreases to a level ≤ 18.0kg/m^2
- Cardiovascular collapse (shock) from whatever cause, acute congestive heart failure, acute myocardial infarction and other conditions characterized by hypoxemia
- Surgical procedures are necessary and can not be replaced by any other treatment
- Any exclusion criteria develops during the course of the study

All subjects who discontinue should comply with protocol specified follow-up procedures as outlined in Section 6. The only exception to this requirement is when a subject withdraws consent for all study procedures or loses the ability to consent freely (ie, is imprisoned or involuntarily incarcerated for the treatment of either a psychiatric or physical illness).

If a subject was withdrawn before completing the study, the reason for withdrawal must be entered on the appropriate case report form (CRF) page.

5 TREATMENTS

5.1 Study Treatment

All protocol-specified investigational and noninvestigational products are considered study drug.

Draft v3.11 930026888

5.1.1 Investigational Product

An investigational product, also known as investigational medicinal product in some regions, is defined as follows:

A pharmaceutical form of an active substance or placebo being tested or used as a reference in a clinical study, including products already with a marketing authorization but used or assembled (formulated or packaged) in a way different from the authorized form, or used for an unauthorized indication, or when used to gain further information about the authorized form.

In this protocol, investigational product(s) is/are:

Glucophage XR 500 mg tablets will be provided with commercial package by Sino-America Shanghai Squibb Pharmaceutical Ltd.

5.1.2 Noninvestigational Product

Other medications used in the study as support or escape medication for preventative, diagnostic, or therapeutic reasons, as components of the standard of care for a given diagnosis, are considered noninvestigational products.

5.1.3 Identification

PRODUCT	POTENCY	APPEARANCE
Glucophage XR tablets	500 mg	white to off-white, capsule shaped, biconvex tablets, with “BMS 6063” debossed on one side and “500” debossed across the face of the other side

5.1.4 Packaging and Labeling

Glucophage XR 500 mg tablets will be provided in a box that contains a bottle of 30 tablets. This product will bear market product labeling for China. And the box will be labeled with a 1-panel auxiliary label that contains information such as: Clinical Research

Draft v3.11 930026888

Only, Not for Sale, Please Return the Unused Drugs and This Package; Protocol number. In addition, a space will also be provided to enter subject number (PID), investigator name and the date of drug dispensing.

5.1.5 Handling and Dispensing

Study drug supplied by the sponsor or sourced by the investigator should be stored in a secure area according to local regulations. It is the responsibility of the investigator to ensure that study drug is only dispensed to study subjects. The study drug must be dispensed only from official study sites by authorized personnel according to local regulations.

The investigator should ensure that the investigational product is stored in accordance with the environmental conditions (temperature, light, and humidity) as determined by the sponsor. Glucophage XR tablets should be stored at 15-25 °C, protected from light and moisture. If concerns regarding the quality or appearance of the investigational product arise, do not dispense the investigational product and contact the sponsor immediately.

Since the visit at Day 1, a supply of Metformin XR will be provided at each visit in amounts sufficient to reach the next study visit. Investigator or designee should fill the PID, the date of drug dispensing and signature at the relative blank of study drug label.

At each visit, subjects will be asked to bring back their bottles (used and unused) of study medication so that drug accountability can be performed and adherence to study drug therapy can be assessed.

Please refer to Section 9.2.2 for information on study drug record retention and 9.3 for return and destruction instructions.

5.2 Method of Assigning Subjects to a Treatment

Each subject who meets the inclusion/exclusion criteria will be assigned to one of three groups: Normal weight, Overweight and Obese based on their baseline BMI in a 1:1:1 ratio. The methods of Glucophage XR treatment will be the same in the three study groups.

Draft v3.11 930026888

The BMI subgroups are defined according to the Chinese guideline 2007: Obese is defined as BMI$\geq$28kg/m^2, overweight is defined as BMI $\geq$24kg/m^2 and $<$28kg/m^2, normal weight is defined as BMI$\geq$18.5kg/m^2 and $<$24kg/m^2.

5.3 Selection and Timing of Dose for Each Subject

Glucophage XR will be administered to subjects enrolled to the study for 16 weeks. The initial dose will be 500mg/day orally taken once daily with the evening meal,

5.3.1 Dose Modifications

Glucophage XR will be up-titrated by increments of 500mg weekly to 1500mg/day unless intolerance or hypoglycemia occurs. At week 4 and afterwards, the maximum daily dose may be 2000mg/day (orally, once daily, with the evening meal), if FPG$>$7.0 mmol/L (126mg/dL).

5.4 Blinding/Unblinding

N/A

5.5 Concomitant Treatments

5.5.1 Prohibited and/or Restricted Treatments

See section 4.2.2.

5.5.2 Other Restrictions and Precautions

- **Concomitant use of cationic drugs (e.g., amiloride, digoxin, morphine, procainamide, quinidine, quinine, ranitidine, triamterene, trimethoprim, or vancomycin):** Careful patient monitoring and dose adjustment of cationic drugs is recommended.
- **Alcohol intake**: Patients should be warned against excessive alcohol intake, acute or chronic, while receiving Glucophage XR.

Draft v3.11 930026888

5.6 Treatment Compliance

Compliance based on study drug pill count will be performed at each scheduled visit from visit 2 for all subjects. Compliance will be reinforced at each study visit.

6 STUDY ASSESSMENTS AND PROCEDURES

6.1 Flow Chart/Time and Events Schedule

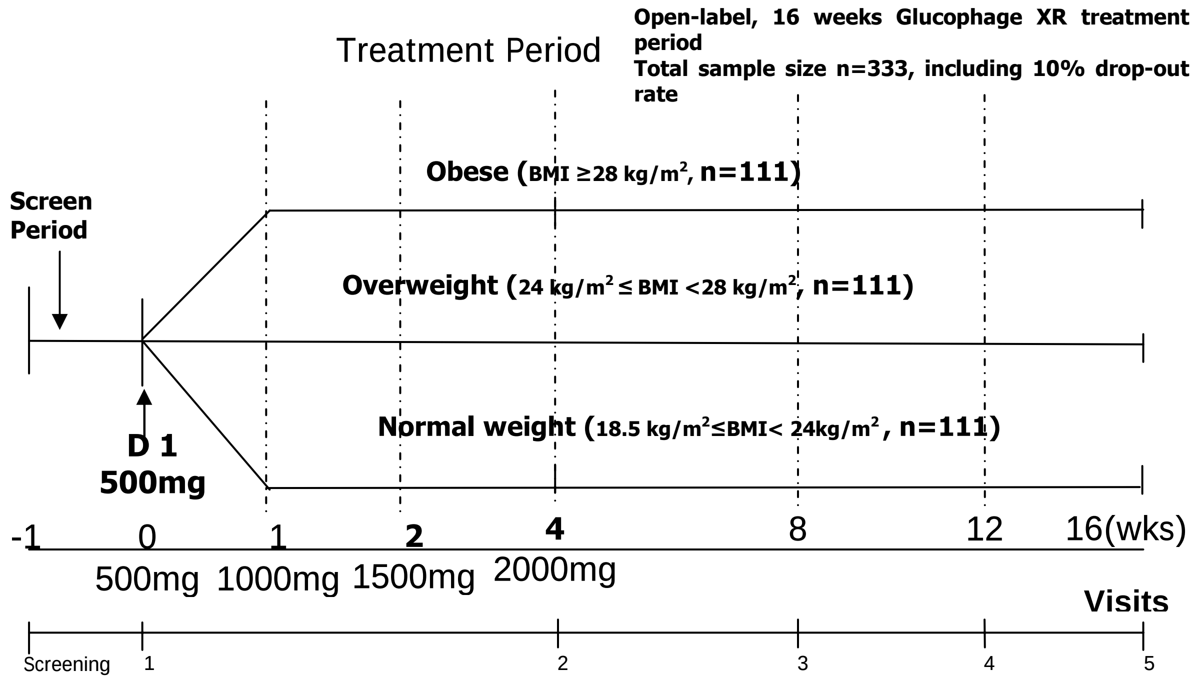

* At week 4, week 8, or week 12, the maximum daily dose may be 2000mg/day (orally, once daily, with the evening meal), if FPG>7.0 mmol/L (126mg/dL).

* If FPG > 10.0 mmol/L (>180 mg/dL) at visits Week 4, Week 8, or Week 12 and confirmed at a repeated measurement in one week, the subject will be discontinued from the treatment.

Draft v3.11 930026888

Table 1: Flow Chart for Protocol CV138097

Procedure	Screening Visit (up to 7 days prior to Day 1)	During Treatment Visit 1 (Day 1)	During Treatment Visit 2 (Week 4± 7days)	During Treatment Visit 3 (Week 8± 7days)	During treatment Visit 4 (Week 12± 7days)	End of Treatment Visit (Week 16± 7days)	Early termination Visit	Protocol Section
**Eligibility Assessments**								
Informed Consent	X							3.3
Inclusion/Exclusion Criteria	X							4.2.1 and 4.2.2
Demography and Medical History	X							6.1
Physical Examination	X					X	X	6.1
Vital Signs	X	X	X	X	X	X	X	6.1
Weight	X	X	X	X	X	X	X	6.1
Height	X							6.1
ECG	X					X	X	6.1
Concomitant medication	X	X	X	X	X	X	X	5.5
**Safety Assessments**								
Adverse Events Assessment		X	X	X	X	X	X	7
Hematology [a]	X					X	X	6.1

Draft v3.11 930026888

Table 1: Flow Chart for Protocol CV138097

Procedure	Screening Visit (up to 7 days prior to Day 1)	During Treatment Visit 1 (Day 1)	During Treatment Visit 2 (Week 4± 7days)	During Treatment Visit 3 (Week 8± 7days)	During treatment Visit 4 (Week 12± 7days)	End of Treatment Visit (Week 16± 7days)	Early termination Visit	Protocol Section
Serum chemistry [a]	X					X	X	6.1
Urinalysis [a]	X					X	X	6.1
Pregnancy test [a]	X					X	X	6.1
**Efficacy Assessments**								
FPG [a]	X	X	X	X	X	X	X	6.1
HbA1c [b]	X					X	X	6.1
Serum Lipids (TC, LDL-C, HDL-C, TG) [a]		X				X	X	6.1
CRP [c]		X				X	X	6.1
PAI-1 [c]		X				X	X	6.1
Adiponectin [c]		X				X	X	6.1
Waist circumference		X				X	X	6.1
Hip circumference		X				X	X	6.1

Draft v3.11 930026888

Table 1: Flow Chart for Protocol CV138097

Procedure	Screening Visit (up to 7 days prior to Day 1)	During Treatment Visit 1 (Day 1)	During Treatment Visit 2 (Week 4± 7days)	During Treatment Visit 3 (Week 8± 7days)	During treatment Visit 4 (Week 12± 7days)	End of Treatment Visit (Week 16± 7days)	Early termination Visit	Protocol Section
**Clinical Drug Supplies**								
Dispensation of investigational drugs		X	X	X	X			5.1-5.3
Dispensation of patient diary		X	X	X	X			9.2.2
Patient diary review			X	X	X	X	X	9.2.2
Drug accountability			X	X	X	X	X	9.2.2
Drug return			X	X	X	X	X	9.3
**Other study procedures**								
Diet and life-style advice	X	X	X	X	X	X	X	6.1

[a] To be assessed in local lab

[b] To be assessed in central lab

[c] To be assessed in central lab only for the first 111 patients is enrolled in Beijing site

6.1.1 Procedures by visit

The study is divided into 2 periods as follows:

Screening period

Screening period of up to 7 days

Treatment period

Glucophage XR treatment of 16 weeks

6.1.2 Visit windows

The procedures scheduled at each visit may be performed on days other than the nominal days specified in the table 1. The allowed deviation from the nominal visit days are tabulated below:

Table 2: Visit windows

Nominal Visit	Visit Days/Allowed Window
Screening	- 7 days
Day 1	Day 1
Week 4	Week 4 ± 7 days
Week 7	Week 8 ± 7 days
Week 11	Week 12 ± 7 days
Week 16	Week 16 ± 7 days

Draft v3.11 930026888

6.1.3 Screening Period- Enrollment Visit (up to 7 days prior to Day 1):

The investigator or designee will:

- Obtain written informed consent
- Obtain relevant medical history and demography
- Perform physical examination
- Obtain vital signs
- Obtain physical measurements including height, weight
- Obtain 12-lead ECG
- Assess concomitant medication use (within 30 days prior to screening visit)
- Obtain clinical laboratory tests (including Hematology, Serum chemistry, Urinalysis, Pregnancy test)
- Obtain FPG, in local lab
- Obtain HbA1c in central lab
- Determine if subject meets inclusion/exclusion criteria
- Advise diet and life-style

6.1.4 Treatment Period:

6.1.4.1 Day 1 Visit:

The investigator or designee will:

- Obtain vital signs
- Obtain physical measurements including weight, waist and hip circumference
- Assess changes in concomitant medication use
- Obtain FPG, serum lipids (TC, LDL-C, HDL-C and TG) in local lab; and CRP, PAI-1 and Adiponectin in central lab only for the first 111 patients enrolled by sites in Beijing
- Dispensation of investigational drugs
- Dispensation of patient diary
- Administer investigational drugs
- Assess AEs and SAEs
- Advise diet and life-style

6.1.4.2 Week 4, 8 and 12 Visits (Week 4, 8 and 12± 7 days):

The investigator or designee will:

- Obtain vital signs
- Obtain physical measurements including Weight
- Assess changes in concomitant medication use
- Obtain FPG
- Dispensation of investigational drugs
- Dispensation of patient diary
- Administer investigational drugs and modify dosage
- Review patient diary
- Assess AEs and SAEs
- Advise diet and life-style
- Drug accountability

6.1.4.3 Week 16 Visit (Week 16 ± 7days) or Early termination Visit:

The investigator or designee will:

- Perform physical examination
- Obtain vital signs
- Obtain physical measurements including height, weight, waist and hip circumference
- Obtain 12-lead ECG
- Assess changes in concomitant medication use
- Obtain clinical laboratory tests (including Hematology, Serum chemistry, Urinalysis, Pregnancy test)
- Obtain FPG, serum lipids (TC, LDL-C, HDL-C and TG) in local lab; HbA1c in central lab; and CRP, PAI-1 and Adiponectin in central lab only for the first 111 patients enrolled by sites in Beijing
- Review patient diary
- Assess AEs and SAEs
- Advise diet and life-style
- Drug accountability

Draft v3.11 930026888

6.2 Study Materials

The following study supplies will be provided

- Paper Case Report Forms
- Sample source document worksheets
- Diary card

6.3 Safety Assessments

6.3.1 Lactic acidosis:

Lactic acidosis is characterized by elevated blood lactate levels (>5 mmol/L), decreased blood pH, electrolyte disturbances with an increased anion gap, and an increased lactate/pyruvate ratio. When metformin is implicated as the cause of lactic acidosis, metformin plasma levels >5 μg/mL are generally found.

The onset of lactic acidosis often is subtle, and accompanied only by nonspecific symptoms such as malaise, myalgias, respiratory distress, increasing somnolence, and nonspecific abdominal distress. There may be associated hypothermia, hypotension, and resistant bradyarrhythmias with more marked acidosis. The subject and the investigator must be aware of the possible importance of such symptoms and the subject should be instructed to notify the investigator immediately if they occur. Glucophage XR should be withdrawn until the situation is clarified. Serum electrolytes, ketones, blood glucose, and if indicated, blood pH, lactate levels, and even blood metformin levels should be assessed. Once a subject is stabilized on any dose level of Glucophage XR, gastrointestinal symptoms, which are common during initiation of therapy, are unlikely to be drug related. Later occurrence of gastrointestinal symptoms could be due to lactic acidosis or other serious disease. Lactic acidosis should be suspected in any diabetic patient with metabolic acidosis lacking evidence of ketoacidosis (ketonuria and ketonemia).

Lactic acidosis is a medical emergency that must be treated in a hospital setting. In a patient with lactic acidosis who is taking Glucophage XR, the drug should be discontinued immediately and general supportive measures promptly instituted. Because

Draft v3.11 930026888

metformin hydrochloride is dialyzable (with a clearance of up to 170 mL/min under good hemodynamic conditions), prompt hemodialysis is recommended to correct the acidosis and remove the accumulated metformin. Such management often results in prompt reversal of symptoms and recovery.

6.3.2 Laboratory Assessments:

Blood and urine samples will be obtained on visits for clinical laboratory evaluations as outlined in Section 6.1. The HbA1c at Screening visit and Week 16 or Early Termination Visit will be tested in Central Lab. The CRP, PAI-1 and Adiponectin will be analyzed in Central Lab. Other lab tests, such as hematology, chemistry (including serum lipid), FPG, urinalysis and pregnancy test, will be evaluated in local lab..

The following laboratory tests are required for this study:

Hematology Profile:

- Hematocrit
- Hemoglobin
- Red Blood Cell Count
- MCV
- MCHC
- MCH
- White Blood Cell Count
- Lymphocytes
- Monocytes
- Basophils
- Eosinophils
- Neutrophils
- Platelet Count

Serum Chemistry

- Albumin
- BUN (Urea)
- Calcium

- Chloride
- Bicarbonate
- CK
- Creatinine
- Glucose
- Potassium
- Sodium
- ALP
- ALT
- AST
- Direct Bilirubin
- Total Bilirubin
- GGT
- Phosphate
- Total Protein
- Uric acid (Urate)
- LDH

Fasting Serum Lipid Profile

- Total Cholesterol
- LDL-C
- HDL-C
- TG

Urinalysis:

- Protein
- Glucose
- Leukocyte Esterase
- Nitrite
- Blood
- pH
- Ketones

Draft v3.11 930026888

- Specific Gravity
- Bilirubin
- Urobilinogen

Pregnancy Tests:

A urine pregnancy test (for WOCBP) to be conducted:

- At screening
- At the Week 16 Visit

6.3.3 Vital Signs:

Vital signs (blood pressure and heart rate) will be recorded during all the visits.

6.3.4 Electrocardiograms:

A 12-lead ECG will be recorded at screening visit and Week 16 visit or Early Termination visit.

6.3.5 Physical Examinations:

Physical examinations will be performed at all the visits.

6.3.6 Physical Measurements:

Height will be measured at screening visit, waist and hip circumference will be measured at the Day 1 and the week 16 or Early Termination visit. Weight will be measured at all the visits.

6.3.7 Concomitant Medications:

At the screening visit, medications that subjects have used in the past 30 days will be recorded. At all other studies, the changes in concomitant medications since the last data collection will also be recorded.

Draft v3.11 930026888

6.4 Efficacy Assessments

6.4.1 Primary Efficacy Assessment

The primary efficacy endpoint of this study is HbA1c change from baseline after a 16-week oral administration of Glucophage XR.

HbA1c will be obtained at screen visit (also as the baseline) and the Week 16 visit or Early Termination Visit.

6.4.2 Secondary Efficacy Assessments

Secondary endpoints are the change from baseline in fasting plasma glucose and the relative changes from baseline in fasting lipids (Total-C, LDL-C, HDL-C and TG).

Fasting plasma glucose will be obtained at all the visits.

Plasma fasting lipids (including TC, LDL-c, HDL-C and TG), CRP, PAI-1 and Adiponectin will be obtained at the Day 1 (as baseline) visit and the week 16 visit or Early Termination Visit.

6.5 Pharmacodynamics Assessments

Not applicable.

6.6 Pharmacogenomics Assessments

Not applicable.

6.7 Outcomes Research Assessments

Not applicable.

6.8 Other Assessments

Not applicable

7 ADVERSE EVENTS

7.1 Definitions

An ***Adverse Event (AE)*** is defined as any new untoward medical occurrence or worsening of a pre-existing medical condition in a patient or clinical investigation subject administered an investigational (medicinal) product and that does not necessarily have a causal relationship with this treatment. An AE can therefore be any unfavorable and unintended sign (including an abnormal laboratory finding, for example), symptom, or disease temporally associated with the use of investigational product, whether or not considered related to the investigational product.

7.1.1 Serious Adverse Events

A ***serious AE (SAE)*** is any untoward medical occurrence that at any dose:

- results in death
- is life-threatening (defined as an event in which the subject was at risk of death at the time of the event; it does not refer to an event which hypothetically might have caused death if it were more severe)
- requires inpatient hospitalization or causes prolongation of existing hospitalization (see note below for exceptions)
- results in persistent or significant disability/incapacity
- is a congenital anomaly/birth defect (note: reports of congenital anomalies/birth defects must also be reported on the Pregnancy Surveillance Form [see Section 7.6])
- is an important medical event (defined as a medical event(s) that may not be immediately life-threatening or result in death or hospitalization but, based upon appropriate medical and scientific judgment, may jeopardize the subject or may require intervention [eg, medical, surgical] to prevent one of the other serious outcomes listed in the definition above.) Examples of such events include, but are not limited to, intensive treatment in an emergency room or at home for allergic bronchospasm; blood dyscrasias or convulsions that do not result in hospitalization.)

All pregnancies, regardless of outcome, must be reported to the sponsor on a Pregnancy Surveillance Form, not an SAE form (see Section 7.6). Although overdose and cancer are

Draft v3.11 930026888

not always serious by regulatory definition, these events should be reported on an SAE form and sent to BMS in an expedited manner.

NOTE:

The following hospitalizations are not considered SAEs in BMS clinical studies:

- a visit to the emergency room or other hospital department < 24 hours, that does not result in admission (unless considered "important medial event" or event life threatening)
- elective surgery, planned prior to signing consent
- admissions as per protocol for a planned medical/surgical procedure
- routine health assessment requiring admission for baseline/trending of health status (eg, routine colonoscopy)
- medical/surgical admission for purpose other than remedying ill health state and was planned prior to entry into the study. Appropriate documentation is required in these cases
- admission encountered for another life circumstance that carries no bearing on health status and requires no medical/surgical intervention (eg, lack of housing, economic inadequacy, care-giver respite, family circumstances, administrative)

7.1.2 Nonserious Adverse Events

All AEs that are not classified as serious.

7.2 Assignment of Adverse Event Intensity and Relationship to Study Drug

The following categories and definitions of intensity as determined by a physician should be used for all BMS clinical study AEs:

- Mild (Grade 1) - Awareness of event but easily tolerated
- Moderate (Grade 2) - Discomfort enough to cause some interference with usual activity
- Severe (Grade 3) - Inability to carry out usual activity
- Very Severe (Grade 4) - Debilitating, significantly incapacitates subject despite symptomatic therapy

Draft v3.11 930026888

The following categories and definitions of causal relationship to study drug as determined by a physician should be used for all BMS clinical study AEs:

- Certain: There is a reasonable causal relationship between the investigational product and the AE. The event responds to withdrawal of investigational product (dechallenge), and recurs with rechallenge when clinically feasible.
- Probable: There is a reasonable causal relationship between the investigational product and the AE. The event responds to dechallenge. Rechallenge is not required.
- Possible: There is reasonable causal relationship between the investigational product and the AE. Dechallenge information is lacking or unclear.
- Not likely: There is a temporal relationship to investigational product administration, but there is not a reasonable causal relationship between the investigational product and the AE.
- Not related: There is not a temporal relationship to investigational product administration (too early, or late, or investigational product not taken), or there is a reasonable causal relationship between noninvestigational product, concurrent disease, or circumstance and the AE.

The expression "reasonable causal relationship" is meant to convey in general that there are facts (eg, evidence such as de-challenge/re-challenge) or other arguments to suggest a positive causal relationship.

7.3 Collection and Reporting

Adverse events can be spontaneously reported or elicited during open-ended questioning, examination, or evaluation of a subject. (In order to prevent reporting bias, subjects should not be questioned regarding the specific occurrence of one or more AEs.)

If known, the diagnosis of the underlying illness or disorder should be recorded, rather than its individual symptoms. The following information should be captured for all AEs: onset, duration, intensity, seriousness, relationship to study drug, action taken, and treatment required. If treatment for the AE was administered, it should be recorded on the appropriate CRF page. The investigator shall supply the sponsor and Ethics Committee with any additional requested information, notably for reported deaths of subjects.

Draft v3.11 930026888

Completion of supplemental CRFs may be requested for AEs and/or laboratory abnormalities that are reported/identified during the course of the study.

7.3.1 Serious Adverse Events

Following the subject's written consent to participate in the study, all SAEs must be collected, including those thought to be associated with protocol-specified procedures. All SAEs must be collected that occur within 30 days of discontinuation of dosing of study drug. If applicable, SAEs must be collected that relate to any later protocol-specified procedure (eg, a follow-up skin biopsy). The investigator should notify BMS of any SAE occurring after this time period is believed to be related to the study drug or protocol-specified procedure.

Serious adverse events, whether related or unrelated to study drug, must be recorded on the SAE page of the CRF and reported within 24 hours to BMS (or designee) to comply with regulatory requirements. An SAE report should be completed for any event where doubt exists regarding its status of seriousness.

All SAEs must be reported within 24 hours by confirmed facsimile transmission (fax) and mailing of the completed SAE page (top, white, original). In some instances where a facsimile machine is not available, overnight express mail may be used. If only limited information is initially available, follow-up reports are required. (Note: Follow-up SAE reports should include the same investigator term(s) initially reported.) In selected circumstances, the protocol may specify conditions that require additional telephone reporting. The electronic SAE CRF in the electronic data capture tool should not be used.

If the investigator believes that an SAE is not related to the study drug, but is potentially related to the conditions of the study (such as withdrawal of previous therapy, or a complication of a study procedure), the relationship should be specified in the narrative section of the SAE page of the CRF.

If an ongoing SAE changes in its intensity or relationship to the study drug, a follow-up SAE report should be sent within 24 hours to the sponsor. As follow-up information becomes available it should be sent within 24 hours using the same procedure used for transmitting the initial SAE report. All SAEs should be followed to resolution or stabilization.

SAE MAILING ADDRESS:

Rui Liu
6F Fuxing Plaza,
109 Yandang Road,
Shanghai 200020, P.R.China

SAE TELEPHONE CONTACT

Name:	Hongmei Li
Office:	86-21-23218293
24 Hour:	86-13636616736
Name:	Iris Bian
Office:	86-21-23218125
24 Hour:	86-15801999540
Name:	Liyong Ren
Office:	86-21-53834000 ext. 118
24 Hour:	86- 86 13817061932

7.3.2 Handling of Expedited Safety Reports

In accordance with local regulations, BMS will notify investigators of all SAEs that are suspected (related to the investigational product) and unexpected (ie, not previously described in the Investigator Brochure). In the European Union (EU), an event meeting these criteria is termed a Suspected, Unexpected Serious Adverse Reaction (SUSAR). Investigator notification of these events will be in the form of an expedited safety report (ESR).

Other important findings which may be reported by the sponsor as an ESR include: increased frequency of a clinically significant expected SAE, an SAE considered associated with study procedures that could modify the conduct of the study, lack of efficacy that poses significant hazard to study subjects, clinically significant safety finding from a nonclinical (eg, animal) study, important safety recommendations from a

Draft v3.11 930026888

study data monitoring committee, or sponsor decision to end or temporarily halt a clinical study for safety reasons.

Upon receiving an ESR from BMS, the investigator must review and retain the ESR with the Investigator Brochure. Where required by local regulations or when there is a central IRB/IEC for the study, the sponsor will submit the ESR to the appropriate IRB/IEC. The investigator and IRB/IEC will determine if the informed consent requires revision. The investigator should also comply with the IRB/IEC procedures for reporting any other safety information.

In addition, suspected serious adverse reactions (whether expected or unexpected) shall be reported by BMS to the relevant competent health authorities in all concerned countries according to local regulations (either as expedited and/or in aggregate reports).

7.3.3 Nonserious Adverse Events

The collection of nonserious AE information should begin at initiation of study drug. Nonserious AE information should also be collected from the start of a placebo lead-in period or other observational period intended to establish a baseline status for the subjects.

If an ongoing nonserious AE worsens in its intensity or its relationship to the study drug changes, a new nonserious AE entry for the event should be completed. Nonserious AEs should be followed to resolution or stabilization, or reported as SAEs if they become serious (see Section 7.3.1). Follow-up is also required for nonserious AEs that cause interruption or discontinuation of study drug, or those that are present at the end of study treatment as appropriate.

All identified nonserious AEs must be recorded and described on the appropriate non serious AE page of the CRF (paper or electronic).

7.4 Laboratory Test Abnormalities

All laboratory test values captured as part of the study should be recorded on the appropriate laboratory test results pages of the CRF, or be submitted electronically from a central laboratory. In addition, the following laboratory abnormalities should also be

Draft v3.11 930026888

captured on the nonserious AE CRF Page (paper or electronic) or SAE paper CRF page as appropriate:

- Any laboratory test result that is clinically significant or meets the definition of an SAE
- Any laboratory abnormality that required the subject to have the study drug discontinued or interrupted
- Any laboratory abnormality that required the subject to receive specific corrective therapy

It is expected that wherever possible, the clinical, rather than the laboratory term would be used by the reporting investigator (eg, anemia versus low hemoglobin value).

7.5 Overdose

All occurrences of overdose must be reported as an SAE (see Section 7.3.1 for reporting details).

An overdose is defined as the accidental or intentional ingestion or infusion of any dose of a product that is considered both excessive and medically important. All occurrences of overdose must be reported as an SAE (see Section 7.3.1 for reporting details.)

Overdose of metformin hydrochloride has occurred, including ingestion of amounts greater than 50 grams. Hypoglycemia was reported in approximately 10% of cases, but no causal association with metformin hydrochloride has been established. Lactic acidosis has been reported in approximately 32% of metformin overdose cases. Metformin is dialyzable with a clearance of up to 170 mL/min under good hemodynamic conditions. Therefore, hemodialysis may be useful for removal of accumulated drug from patients in whom metformin overdosage is suspected.

7.6 Pregnancy

Sexually active WOCBP must use an effective method of birth control during the course of the study, in a manner such that risk of failure is minimized (See Section 4.2.1 for the definition of WOCBP).

Draft v3.11 930026888

Before enrolling WOCBP in this clinical study, investigators must review the sponsor-provided information about study participation for WOCBP. The topics include the following:

- General Information
- Informed Consent Form
- Pregnancy Prevention Information Sheet
- Drug Interactions with Hormonal Contraceptives
- Contraceptives in Current Use
- Guidelines for the Follow-up of a Reported Pregnancy

Prior to study enrollment, WOCBP must be advised of the importance of avoiding pregnancy during study participation and the potential risk factors for an unintentional pregnancy. The subject must sign an informed consent form documenting this discussion.

7.6.1 Requirements for Pregnancy Testing

All WOCBP MUST have a **negative** pregnancy test within 72 hours as specified in Section 6.1 **prior** to receiving investigational product. The minimum sensitivity of the pregnancy test must be 25 IU/L or equivalent units of HCG. If the pregnancy test is positive, the subject must not receive the investigational product and must not continue in the study.

Pregnancy testing must also be performed throughout the study as specified in Section 6.1 (see flow chart/time and events schedule) and the results of all pregnancy tests (positive or negative) recorded on the CRF or transferred electronically.

In addition, all WOCBP should be instructed to contact the investigator immediately if they suspect they might be pregnant (eg, missed or late menstrual period) at any time during study participation.

Although WOCBP are excluded from participation in this study, some pregnancy testing may be required. Pregnancy testing must be performed throughout the study as specified in Section 6.1 and the results of all pregnancy tests (positive or negative) recorded on the CRF or transferred electronically. The minimum sensitivity of the pregnancy test must be

25 IU/L or equivalent units of HCG. If the pregnancy test is positive, the subject must not receive the investigational product and must not continue in the study.

In addition, all WOCBP should be instructed to contact the investigator immediately if they suspect they might be pregnant (eg, missed or late menstrual period) at any time during study participation.

7.6.2 Reporting of Pregnancy

If, following initiation of investigational product, it is subsequently discovered that a study subject is pregnant or may have been pregnant at the time of investigational product exposure, including during at least 6 half-lives after product administration, the investigational product will be permanently discontinued in an appropriate manner (eg, dose tapering if necessary for subject safety). Exceptions to investigational product discontinuation may be considered for life-threatening conditions only after consultation with the BMS medical monitor or as otherwise specified in this protocol. The investigator must immediately notify the BMS medical monitor of this event, record the pregnancy on the Pregnancy Surveillance Form. Initial information on a pregnancy must be reported immediately to BMS and the outcome information provided once the outcome is known. Forward these forms to BMS according to SAE reporting procedures described in Section 7.3.1.

Protocol-required procedures for study discontinuation and follow-up must be performed on the subject unless contraindicated by pregnancy (eg, x-ray studies). Other appropriate pregnancy follow-up procedures should be considered if indicated. Follow-up information regarding the course of the pregnancy, including perinatal and neonatal outcome must be reported on the Pregnancy Surveillance Form. Infants should be followed for a minimum of 8 weeks.

7.7 Other Safety Considerations

Any significant worsening noted during interim or final physical examinations, electrocardiograms, x-rays, and any other potential safety assessments, whether or not these procedures are required by the protocol, should also be recorded on the appropriate AE page of the CRF (paper or electronic) or SAE paper CRF page.

Draft v3.11 930026888

8 STATISTICAL CONSIDERATIONS

8.1 Sample Size Determination

The sample size is geared to allow estimating mean change from baseline in HbA1c at Week 16 with sufficient precision in each of the 3 subgroups according to baseline BMI. Assuming the standard deviation for the changes from baseline in HbA1c maximally is 1.0 across the baseline BMI subgroups envisaged, 97 patients for a single subgroup will be sufficient to estimate the mean change in HbA1c with a precision of 0.20% within the subgroup. Given the number of baseline BMI subgroups and given that no correction for reason of multiplicity will be made to the 95% confidence level within each BMI subgroup, the total sample size will be 291 for this study. The sample size calculation was performed using the method "confidence interval for mean for one group" available in the nQuery Advisor v6.0 statistical software.

8.2 Populations for Analyses

The Last Observation Carried Forward (LOCF) data set includes data recorded at a given visit or, if no observation is recorded at that visit, data carried forward from the previous visit. Baseline data will not be carried forward or be averaged with on-treatment data to impute missing values for the LOCF data set.

The Observed Case (OC) data set consists of the actual observations at each visit. Efficacy analyses will be performed using both the LOCF and OC data sets. The LOCF data set is the primary data set for the Efficacy Analyses.

8.3 Endpoint Definitions

The primary efficacy variable will be the change from baseline in HbA1c at Week16.

Secondary efficacy variables will be the change from baseline in fasting plasma glucose, the relative changes from baseline in fasting lipids, and the changes from baseline in CRP, PAI-I, and adinopectin.

Draft v3.11 930026888

8.4 Analyses

All analyses will be presented using BMI subgroups defined according to the Chinese guidelines:

- Obese is defined as baseline BMI ≥ 28 kg/m^2
- Overweight is defined as baseline BMI ≥ 24 kg/m^2 and < 28 kg/m^2

Normal weight is defined as baseline BMI ≥ 18.5 kg/m^2 and < 24 kg/m^2.

8.4.1 Demographics and Baseline Characteristics

Patients' demographic and baseline disease characteristics will be summarized by baseline BMI subgroup and overall for both the All Enrolled Subjects Sample and the All Treated Subjects Sample. For continuous variables, descriptive statistics will include number observed, mean and median, standard deviation and inter-quartile range, and extremes. For categorical variables, frequency distributions will be provided.

8.4.2 Safety Analyses

All safety analyses will be performed on the All Treated Subjects Sample. AEs will be classified by Primary System Organ Class (SOC) and Preferred Term (PT) according to the Medical Dictionary for Regulatory Activities (MedDRA).

The incidence of treatment-emergent adverse events will be tabulated by baseline BMI subgroup as well as overall. In addition, the incidence of treatment-emergent AEs considered related to study medication (ie, those AEs judged by the investigator to be either certainly, probably or possibly related to study medication and those with missing causal relationship) as well as the incidence of treatment-emergent AEs with severe or very severe intensity will be reported by baseline BMI subgroup and overall.

All SAEs and those AEs leading to permanent discontinuation of study medication will be reported by baseline BMI subgroup and overall. Special attention will also be paid to reporting cases of lactic acidosis and hypoglycemia.

Draft v3.11 930026888

The incidence of clinically relevant changes observed at any time during treatment versus baseline will be reported by BMI baseline subgroup and overall for clinically relevant increases in body weight (i.e., increase of at least 7% from baseline), for clinically relevant decreases in body weight (i.e., decrease of at least 7% from baseline), for clinically relevant increases in BMI (i.e., increase of at least 1 kg/m^2 from baseline), and for clinically relevant decreases in BMI (i.e., decrease of at least 1 kg/m^2 from baseline)

The incidence of clinically relevant changes observed at any time during treatment versus baseline in safety-related laboratory analytes, in ECG findings, and in vital signs will be reported by baseline BMI subgroup and overall.

In addition, summary statistics will be provided by baseline BMI subgroup and overall for body weight, BMI, waist circumference, vital signs, ECG parameters, and safety-related laboratory analytes. Summaries will be provided per study week assessments were planned and will consist of the number of patients assessed, sample mean value observed together with its associated standard error, sample mean change versus baseline together with its associated standard error and 95% confidence interval.

8.4.3 Efficacy Analyses

All efficacy analyses will be performed on the All Treated Subjects Sample.

Summary statistics will be provided for the primary and all secondary efficacy variables overall as well as for each of the baseline BMI subgroups.

Analysis of Variance (ANOVA) will be used with change from baseline in HbA1c at Week 16 as dependent variable and BMI subgroup (with categories "normal weight", "obese", and "overweight" defined on the basis of baseline BMI according to the Chinese guidelines) as main effect will be applied. The latter ANOVA analysis will be employed for contrasting the treatment effect in the normal weight subgroup versus the other two baseline BMI subgroups. Both contrasts will be interpreted at a two-sided 5% significance level, without correction for multiplicity.

An Analysis of Covariance (ANCOVA) model with change from baseline in fasting plasma glucose as dependent variable, BMI subgroup (with 3 categories as defined above) as main effect, and baseline fasting plasma glucose as covariate will be performed

to examine the relationship between baseline BMI and fasting plasma glucose reduction. To examine the relationship between baseline BMI and relative changes in fasting lipids, similar ANCOVA models will be applied on log-transformed fasting lipids values.

To study the relationship between baseline BMI and change from baseline in HbA1c, and to assess potential predictive factors influencing response to Metformin monotherapy (Glucophage XR), a multiple regression model with change from baseline in HbA1c at Week 16 as dependent variable will be performed. The covariates that will be used in the analysis are age at diagnosis of diabetes, duration of diabetes at time of commencing Glucophage XR treatment, gender, baseline HbA1c, baseline waist/hip ratio, and baseline BMI..

Summary statistics will be provided per study week assessments were planned and will consist of number of patients assessed, sample mean value observed together with its associated standard error, sample mean change observed together with its associated standard error and 95% confidence interval. To summarize relative changes from baseline in fasting lipids [total cholesterol, low-density lipoprotein cholesterol (LDL-C), high-density lipoprotein cholesterol (HDL-C), and triglycerides (TG)], a log-transformation will be applied on the raw data prior to calculating summary statistics. Estimates of mean changes and associated standard errors and 95% confidence intervals calculated on the log scale will be back-transformed into the original scale.

8.4.4 Pharmacokinetic Analyses

Not applicable.

8.4.5 Pharmacodynamic Analyses

Not applicable.

8.4.6 Pharmacogenomic Analyses

Not applicable.

Draft v3.11 930026888

8.4.7 Outcomes Research Analyses

Not applicable.

8.4.8 Other Analyses

Not applicable.

8.5 Interim Analyses

Not applicable.

9 ADMINISTRATIVE SECTION

9.1 Compliance

9.1.1 Compliance with the Protocol and Protocol Revisions

The study shall be conducted as described in this approved protocol. All revisions to the protocol must be discussed with, and be prepared by, BMS. The investigator should not implement any deviation or change to the protocol without prior review and documented approval/favorable opinion from the IRB/IEC of an amendment, except where necessary to eliminate an immediate hazard(s) to study subjects. Any significant deviation must be documented in the CRF.

If a deviation or change to a protocol is implemented to eliminate an immediate hazard(s) prior to obtaining IRB/IEC approval/favorable opinion, as soon as possible the deviation or change will be submitted to:

- IRB/IEC for review and approval/favorable opinion
- Bristol-Myers Squibb
- Regulatory Authority(ies), if required by local regulations

Documentation of approval signed by the chairperson or designee of the IRB(s)/IEC(s) must be sent to BMS.

If an amendment substantially alters the study design or increases the potential risk to the subject: (1) the consent form must be revised and submitted to the IRB(s)/IEC(s) for review and approval/favorable opinion; (2) the revised form must be used to obtain consent from subjects currently enrolled in the study if they are affected by the amendment; and (3) the new form must be used to obtain consent from new subjects prior to enrollment.

If the revision is an administrative letter, investigators must inform their IRB(s)/IEC(s).

9.1.2 Monitoring

Representatives of BMS must be allowed to visit all study site locations periodically to assess the data quality and study integrity. On site they will review study records and directly compare them with source documents, and discuss the conduct of the study with the investigator, and verify that the facilities remain acceptable.

In addition, the study may be evaluated by BMS internal auditors and government inspectors who must be allowed access to CRFs, source documents, other study files, and study facilities. BMS audit reports will be kept confidential.

THE INVESTIGATOR MUST NOTIFY BMS PROMPTLY OF ANY INSPECTIONS SCHEDULED BY REGULATORY AUTHORITIES, AND PROMPTLY FORWARD COPIES OF INSPECTION REPORTS TO BMS.

9.1.3 Investigational Site Training

Bristol-Myers Squibb will provide quality investigational staff training prior to study initiation. Training topics will include but are not limited to: GCP, AE reporting, study details and procedure, study documentation, informed consent, and enrollment of WOCBP.

9.2 Records Retention

The investigator must retain study drug (those supplied by the sponsor or sourced by the investigator) disposition records, copies of CRFs (or electronic files), and source documents for the maximum period required by applicable regulations and guidelines, or

Draft v3.11 930026888

institution procedures, or for the period specified by the sponsor, whichever is longer. The investigator must contact BMS prior to destroying any records associated with the study.

BMS will notify the investigator when the study records are no longer needed.

If the investigator withdraws from the study (eg, relocation, retirement), the records shall be transferred to a mutually agreed upon designee (eg, another investigator, IRB). Notice of such transfer will be given in writing to BMS.

9.2.1 Case Report Forms

An investigator is required to prepare and maintain adequate and accurate case histories designed to record all observations and other data pertinent to the investigation on each individual treated or entered as a control in the investigation. Data reported on the CRF that are derived from source documents must be consistent with the source documents or the discrepancies must be explained.

Paper CRFs must be completed legibly in ink. Subjects are to be identified by birth date and subject number, if applicable. All requested information must be entered on the CRF in the spaces provided. If an item is not available or is not applicable, it must be documented as such; do not leave a space blank

The confidentiality of records that could identify subjects must be protected, respecting the privacy and confidentiality rules in accordance with the applicable regulatory requirement(s).

The investigator will maintain a signature sheet to document signatures and initials of all persons authorized to make entries and/or corrections on CRFs.

For paper CRFs, a correction must be made by striking through the incorrect entry with a single line and entering the correct information adjacent to the incorrect entry. The correction must be dated, initialed and explained (if necessary) by the person making the correction and must not obscure the original entry.

Draft v3.11 930026888

The completed CRF, including any paper SAE/pregnancy CRFs, must be promptly reviewed, signed, and dated by a qualified physician who is an investigator or subinvestigator. The investigator must retain a copy of the CRFs including records of the changes and corrections.

9.2.2 Study Drug Records

9.2.3 Investigational Product Records

It is the responsibility of the investigator to ensure that a current record of investigational product disposition is maintained at each study site where investigational product is inventoried and disposed. Records or logs must comply with applicable regulations and guidelines and should include:

- amount received and placed in storage area
- amount currently in storage area
- label ID number or batch number and use date or expiry date
- dates and initials of person responsible for each investigational product inventory entry/movement
- amount dispensed to and returned by each subject, including unique subject identifiers
- amount transferred to another area/site for dispensing or storage
- non-study disposition (eg, lost, wasted, broken)
- amount returned to the sponsor
- amount destroyed at study site, if applicable
- retain samples sent to third party for bioavailability/bioequivalence, if applicable

The sponsor will provide forms to facilitate inventory control if the staff at the investigational site does not have an established system that meets these requirements.

9.3 Return and Destruction of Study Drug

9.3.1 Return of Study Drug

Upon completion or termination of the study, all unused and/or partially used investigational product must be returned to BMS, if not authorized by BMS to be destroyed at the site.

All investigational product returned to BMS must be accompanied by the appropriate documentation and be clearly identified by protocol number and study site number on the outermost shipping container. Returned supplies should be in the original containers (eg, patient kits that have clinical labels attached). Empty containers should not be returned to BMS. It is the investigator’s responsibility to arrange for disposal of all empty containers, provided that procedures for proper disposal have been established according to applicable federal, state, local, and institutional guidelines and procedures, and provided that appropriate records of disposal are kept. The return of unused investigational product(s) should be arranged by the responsible Study Monitor.

9.3.2 Destruction of Study Drug

If investigational products are to be destroyed on site, it is the investigator’s responsibility to ensure that arrangements have been made for the disposal, written authorization has been granted by BMS, procedures for proper disposal have been established according to applicable regulation and guidelines and institutional procedures, and appropriate records of the disposal have been documented. The unused investigational products can only be destroyed after being inspected and reconciled by the responsible BMS study monitor.

9.4 Publications

The data collected during this study are confidential and proprietary to the sponsor. Any publications or abstracts arising from this study require approval by the sponsor prior to publication or presentation and must adhere to the sponsor’s publication requirements as set forth in the approved clinical trial agreement (CTA). All draft publications, including abstracts or detailed summaries of any proposed presentations, must be submitted to the

Draft v3.11 930026888

sponsor at the earliest practicable time for review, but at any event not less than 30 days before submission or presentation unless otherwise set forth in the CTA. Sponsor shall have the right to delete any confidential or proprietary information contained in any proposed presentation or abstract and may delay publication for up to 60 days for purposes of filing a patent application.

Draft v3.11 930026888

10 GLOSSARY OF TERMS

Term	Definition
Adverse Event	Any new untoward medical occurrence or worsening of a pre-existing medical condition in a patient or clinical investigation subject administered an investigational (medicinal) product and that does not necessarily have a causal relationship with this treatment. An adverse event can therefore be any unfavorable and unintended sign (including an abnormal laboratory finding, for example), symptom, or disease temporally associated with the use of investigational product, whether or not considered related to the investigational product.
Adverse Reaction	An adverse event that is considered by either the investigator or the sponsor as certainly, probably, or possibly to the investigational product
Expedited Safety Report	Rapid notification to investigators of all SAEs that are suspected (certainly, probably, or possibly related to the investigational product) and unexpected (ie, not previously described in the Investigator Brochure), or that could be associated with the study procedures.
Serious Adverse Event	Serious adverse event defined as any untoward medical occurrence that at any dose: results in death; is life-threatening (defined as an event in which the subject was at risk of death at the time of the event; it does not refer to an event which hypothetically might have caused death if it were more severe), requires inpatient hospitalization or causes prolongation of existing hospitalization; results in persistent or significant disability/incapacity, is a congenital anomaly/birth defect; is an important medical event (defined as a medical event(s) that may not be immediately life-threatening or result in death or hospitalization but, based upon appropriate medical and scientific judgment, may jeopardize the subject or may require intervention [eg, medical, surgical] to prevent one of the other serious outcomes listed in the definition above.) Examples of such events include, but are not limited to, intensive treatment in an emergency room or at home for allergic bronchospasm; blood dyscrasias or convulsions that do

Draft v3.11 930026888

Term	Definition
	not result in hospitalization.). For reporting purposes only, BMS also considers the occurrence of pregnancy, overdose (regardless of association with an AE), and cancer as important medical events.
SUSAR	Suspected, Unexpected, Serious Adverse Reaction as termed by the European Clinical Trial Directive (2001/20/EC).
Unexpected Adverse Reaction	An adverse reaction, the nature or severity of which is not consistent with the applicable product information (eg, Investigator Brochure for an unapproved investigational product)
Adverse Event	Any new untoward medical occurrence or worsening of a pre-existing medical condition in a patient or clinical investigation subject administered an investigational (medicinal) product and that does not necessarily have a causal relationship with this treatment. An adverse event can therefore be any unfavorable and unintended sign (including an abnormal laboratory finding, for example), symptom, or disease temporally associated with the use of investigational product, whether or not considered related to the investigational product.

11 LIST OF ABBREVIATIONS

Term	Definition
**A**	
ADA	American Diabetes Association
ALP	Alkaline Phosphatase
ALT	Alanine Transaminase
AST	Aspartate Aminotransferase
ANOVA	Analysis of covariance
**B**	
BMI	Body mass index
BMS	Bristol-Myers Squibb
BUN	Blood urea nitrogen
**C**	
CK	Creatine Kinase
CRF	Case report form
CRP	C-reactive protein
CT	Computer tomography
CTA	Clinical Trial Agreements
**D**	
DBP	Diastolic Blood Pressure
**E**	
EASD	European Association for the Study of Diabetes
ECG	Electrocardiogram
**F**	
FSH	Follicle-stimulating hormone
FPG	Fasting plasma glucose
**G**	
GCP	Good Clinical Practice
GGT	Gamma-Glutamyl Transpeptidase
**H**	
HbA1c	Glycosylated haemoglobin A1c
HDL-C	High-density lipoprotein
HCG	Human Chorionic Gonadotropin
HRT	Hormone Replacement Therapy
**I**	
ICH	International Conference on Harmonisation

Draft v3.11 930026888

**Term**	**Definition**
IDF	International Diabetes Federation
IEC	Independent Ethics Committee
IR	Immediate-Release
IRB	Institutional Review Board
IU	International Unit
**L**	
LDH	Lactate Dehydrogenase
LDL-C	Low-density lipoprotein
LOCF	Last observation carried forward
**M**	
MCH	Mean corpuscular hemoglobin
MCHC	Mean corpuscular hemoglobin concentration
MCV	Mean Corpuscular Volume
MedDRA	Medical dictionary for regulatory activities
**N**	
NICE	National Institute for Health and Clinical Excellence
NYHA	New York Heart Association
**O**	
OC	Observed case
**P**	
PAI-1	Plasminogen Activator Inhibitor-1
PID	Patient Identification
PT	Preferred term
**Q**	
QD	*quaque die (Latin)* / daily
**S**	
SBP	Systolic Blood Pressure
SOC	System organ class
**T**	
T2DM	Type 2 diabetes
TC	Total cholesterol
TG	Triglycerides
**U**	
UKPDS	United Kingdom Prospective Diabetes Study
ULN	Upper Limit of Normal
**W**	

Draft v3.11 930026888

Term	Definition
WOCBP	Women of childbearing potential
**X**	
XR	Extended release

Draft v3.11 930026888

12 REFERENCES

1. United Kingdom Prospective Diabetes Study Group. *Effect of intensive blood-glucose control with metformin on complications in overweight patients with type 2 diabetes (UKPDS 34).* Lancet, 1998. **352**(9131): p. 854-65.
2. United Kingdom Prospective Diabetes Study Group. *United Kingdom Prospective Diabetes Study 24: a 6-year, randomized, controlled trial comparing sulfonylurea, insulin, and metformin therapy in patients with newly diagnosed type 2 diabetes that could not be controlled with diet therapy.* Ann Intern Med, 1998. **128**(3): p. 165-75.
3. Nathan, D.M., et al., *Management of hyperglycaemia in type 2 diabetes: a consensus algorithm for the initiation and adjustment of therapy. A consensus statement from the American Diabetes Association and the European Association for the Study of Diabetes.* Diabetologia, 2006. **49**(8): p. 1711-21.
4. *Standards of medical care in diabetes--2007.* Diabetes Care, 2007. **30 Suppl 1**: p. S4-S41.
5. *Global Guideline for Type 2 Diabetes: recommendations for standard, comprehensive, and minimal care.* Diabet Med, 2006. **23**(6): p. 579-93.
6. McIntosh, A., et al., *Clinical guidelines and evidence review for Type 2 diabetes: management of blood glucose, ScHARR, University of Sheffield, Sheffield, 2001.* 2002.
7. *Type 2 diabetes: practical targets and treatments Fourth edition. Asian-Pacific Type 2 Diabetes Policy Group.* 2005.
8. Ong, C.R., et al., *Long-term efficacy of metformin therapy in nonobese individuals with type 2 diabetes.* Diabetes Care, 2006. **29**(11): p. 2361-4.
9. Donnelly, L.A., et al., *The effect of obesity on glycaemic response to metformin or sulphonylureas in Type 2 diabetes.* Diabet Med, 2006. **23**(2): p. 128-33.
10. Lund, S.S., et al., *Targeting hyperglycaemia with either metformin or repaglinide in non-obese patients with type 2 diabetes: results from a randomized crossover trial.* Diabetes Obes Metab, 2007. **9**(3): p. 394-407.
11. Timmins, P., et al., *Steady-state pharmacokinetics of a novel extended-release metformin formulation.* Clin Pharmacokinet, 2005. **44**(7): p. 721-9.

Draft v3.11 930026888

12. Fujioka, K., et al., *Efficacy, dose-response relationship and safety of once-daily extended-release metformin (Glucophage XR) in type 2 diabetic patients with inadequate glycaemic control despite prior treatment with diet and exercise: results from two double-blind, placebo-controlled studies.* Diabetes Obes Metab, 2005. **7**(1): p. 28-39.

13. Fujioka, K., M. Pans, and S. Joyal, *Glycemic control in patients with type 2 diabetes mellitus switched from twice-daily immediate-release metformin to a once-daily extended-release formulation.* Clin Ther, 2003. **25**(2): p. 515-29.

14. Blonde, L., et al., *Gastrointestinal tolerability of extended-release metformin tablets compared to immediate-release metformin tablets: results of a retrospective cohort study.* Curr Med Res Opin, 2004. **20**(4): p. 565-72.

15 Chinese type 2 Diabetes prevention and treatment guideline(2007). Chinese Journal of Endocrinology and Metabolism 2008; 2, **Supplement** 2a:1–22.

APPENDIX 1 ADDITIONAL ETHICAL CONSIDERATIONS

1 INFORMED CONSENT PROCEDURES

BMS will provide the investigator with an appropriate (ie, Global or Local) sample informed consent form which will include all elements required by ICH, GCP and applicable regulatory requirements. The sample informed consent form will adhere to the ethical principles that have their origin in the Declaration of Helsinki. If the investigator makes changes to the informed consent form sample, BMS will ensure all required elements and local regulatory and legal requirements are met.

The consent form must also include a statement that BMS and regulatory authorities have direct access to subject records. Prior to the beginning of the study, the investigator must have the IRB/IEC’s written approval/favorable opinion of the written informed consent form and any other information to be provided to the subjects.

The investigator must provide the subject, or, in those situations where consent cannot be given by subjects, their legally acceptable representative with a copy of the consent form and written information about the study in the language in which the subject is most proficient. The language must be non-technical and easily understood. The investigator should allow time necessary for subject or subject's legally acceptable representative to inquire about the details of the study, then informed consent must be signed and personally dated by the subject or the subject's legally acceptable representative and by the person who conducted the informed consent discussion. The subject or legally acceptable representative should receive a copy of the signed informed consent and any other written information provided to study subjects prior to subject's participation in the study.

1.1 Subjects Unable to Give Written Informed Consent

1.1.1 Minors

For minors, according to local legislation, one or both parents or a legally acceptable representative must be informed of the study procedures and must sign the informed consent form approved for the study prior to clinical study participation. (In the event that

Draft v3.11 930026888

the parents or legal guardians are unable to read, then an impartial witness should be present during the entire informed consent discussion). Whenever feasible, minors who are judged to be of an age of reason must also give their written assent by signing and dating the completed informed consent. All local laws, rules and regulations regarding informed consent of minors must be followed.

1.1.2 Subjects Experiencing Acute Events or Emergencies

A legally acceptable representative or legal guardian must provide informed consent when consent of the subject is not possible prior to clinical study participation, eg, for subjects experiencing an acute medical event such as myocardial infarction or stroke. Informed consent of the subject must additionally be obtained if they become capable of making and communicating their informed consent during the clinical study. All local laws, rules and regulations regarding informed consent of adult subjects incapable of giving informed consent must be followed.

1.1.3 Mentally Impaired or Incapacitated Subjects

Investigators (or whoever required by local regulations) should determine whether or not a mentally impaired or incapacitated subject is capable of giving informed consent and should sign a statement to that effect. If the subject is deemed mentally competent to give informed consent, the investigator should follow standard procedures. If the subject is deemed not to be mentally competent to give informed consent, a fully informed legal guardian or legally acceptable representative can be asked to give consent for, or on behalf of, the subject. All local laws, rules and regulations regarding informed consent of mentally impaired or incapacitated subjects must be followed.

Patients who are involuntarily hospitalized because of mental illness must not be enrolled in clinical studies

1.1.4 Other Circumstances

Subjects who are imprisoned or involuntarily detained for treatment of either a psychiatric or physical (eg, infectious disease) illness must not be enrolled in clinical studies.

In circumstances where a subject's only access to treatment is through enrollment in a clinical study, eg, for subjects in developing countries with limited resources or for subjects with no marketed treatment options, the investigator must take special care to explain the potential risks and benefits associated with the study and ensure that the subject is giving informed consent.

When a subject may be in a dependent relationship with the investigator, a well-informed physician who is not engaged in the clinical study and is completely independent of the relationship between the subject and investigator should obtain the subject's informed consent.

1.1.5 Illiterate Subjects

If the subject, or, in those situations where consent cannot be given by the subject, their legally acceptable representative is unable to read, a reliable and independent witness should be present during the entire informed consent discussion. The choice of the witness must not breach the subject's rights to confidentiality. A reliable independent witness is defined as one not affiliated with the institution or engaged in the investigation. A family member or acquaintance is an appropriate independent witness. After the subject or legally acceptable representative orally consents and has signed, if capable, the witness should sign and personally date the consent form attesting that the information is accurate and that the subject, or, in those situations where consent cannot be given by subjects, their legally acceptable representative has fully understood the content of the informed consent agreement and is giving true informed consent.

1.2 Update of Informed Consent

The informed consent and any other information provided to subjects, or, in those situations where consent cannot be given by subjects, the subject's legally acceptable representative, should be revised whenever important new information becomes available that is relevant to the subject's consent, and should receive IRB/IEC approval/favorable opinion prior to use. The investigator, or a person designated by the investigator should fully inform the subject or the subject's legally acceptable representative of all pertinent aspects of the study and of any new information relevant to the subject's willingness to continue participation in the study. This communication should be documented.

Draft v3.11 930026888

During a subject's participation in the study, any updates to the consent form and any updates to the written information will be provided to the subject.

Draft v3.11 930026888
